# Sulfolane-Based Flame-Retardant Electrolyte for High-Voltage Sodium-Ion Batteries

**DOI:** 10.1007/s40820-024-01546-7

**Published:** 2024-10-18

**Authors:** Xuanlong He, Jie Peng, Qingyun Lin, Meng Li, Weibin Chen, Pei Liu, Tao Huang, Zhencheng Huang, Yuying Liu, Jiaojiao Deng, Shenghua Ye, Xuming Yang, Xiangzhong Ren, Xiaoping Ouyang, Jianhong Liu, Biwei Xiao, Jiangtao Hu, Qianling Zhang

**Affiliations:** 1 Graphene Composite Research Center, College of Chemistry and Environmental Engineering, Shenzhen University, Shenzhen, 518060 People’s Republic of China; 2 Center of Electron Microscopy, State Key Laboratory of Silicon and Advanced Semiconductor Materials, School of Materials Science and Engineering, Zhejiang University, Hangzhou, 310027 People’s Republic of China; 3 GRINM (Guangdong) Research Institute for Advanced Materials and Technology, Foshan, Guangdong 528051 People’s Republic of China; 4 College of Energy Engineering, Zhejiang University, Hangzhou, Zhejiang 310027 People’s Republic of China; 5 School of Materials Science and Engineering, Xiangtan University, Xiangtan, 411105 People’s Republic of China; 6 Shenzhen Eigen-Equation Graphene Technology Co. Ltd, Shenzhen, 518000 People’s Republic of China

**Keywords:** Sodium-ion batteries, Sulfolane-based electrolyte, High voltage, Layered oxide cathode, Flame retardant

## Abstract

Sodium-ion batteries hold great promise as next-generation energy storage systems. However, the high instability of the electrode/electrolyte interphase during cycling has seriously hindered the development of SIBs. In particular, an unstable cathode–electrolyte interphase (CEI) leads to successive electrolyte side reactions, transition metal leaching and rapid capacity decay, which tends to be exacerbated under high-voltage conditions. Therefore, constructing dense and stable CEIs are crucial for high-performance SIBs. This work reports localized high-concentration electrolyte by incorporating a highly oxidation-resistant sulfolane solvent with non-solvent diluent 1H, 1H, 5H-octafluoropentyl-1, 1, 2, 2-tetrafluoroethyl ether, which exhibited excellent oxidative stability and was able to form thin, dense and homogeneous CEI. The excellent CEI enabled the O3-type layered oxide cathode NaNi_1/3_Mn_1/3_Fe_1/3_O_2_ (NaNMF) to achieve stable cycling, with a capacity retention of 79.48% after 300 cycles at 1 C and 81.15% after 400 cycles at 2 C with a high charging voltage of 4.2 V. In addition, its nonflammable nature enhances the safety of SIBs. This work provides a viable pathway for the application of sulfolane-based electrolytes on SIBs and the design of next-generation high-voltage electrolytes.

**Supplementary Information:**

The online version contains supplementary material available at 10.1007/s40820-024-01546-7.

## Highlights

- NaTFSI/SUL:OTE:FEC facilitates the formation of S, N-rich, dense and robust cathode–electrolyte interphase on NaNMF cathode, which improves the cycling stability under high voltage.
- By utilizing NaTFSI/SUL:OTE:FEC, the Na||NaNMF batteries achieved an impressive retention of 81.15% after 400 cycles at 2 C with the cutoff voltage of 4.2 V.
- The study offers a reference for the utilization of sulfolane-based electrolytes in sodium-ion batteries (SIBs), while the nonflammability of the NaTFSI/SUL:OTE:FEC enhances the safety of SIBs.

**Supplementary Information:**

The online version contains supplementary material available at 10.1007/s40820-024-01546-7.

## Graphical abstract

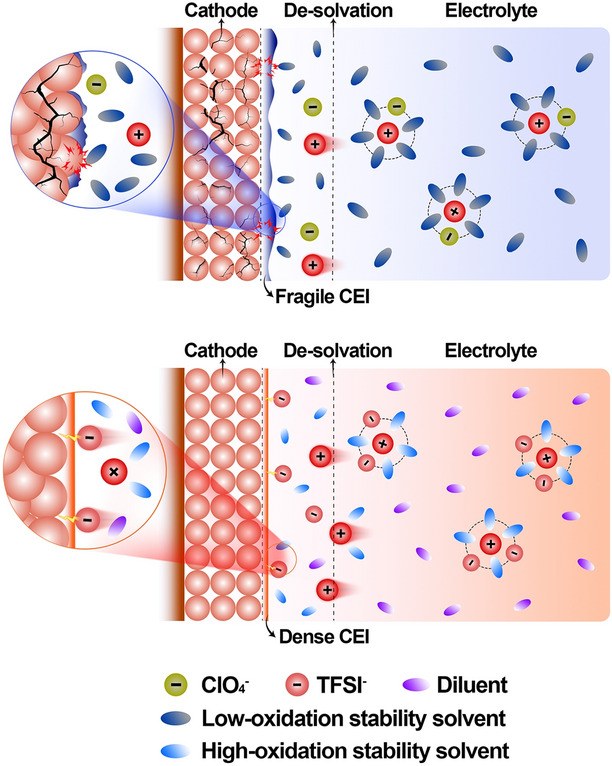

## Introduction

Sodium-ion batteries (SIBs) have become a promising alternative energy source for emerging energy storage devices due to the abundant content of sodium in the earth's crust (23,000 ppm sodium versus 17 ppm lithium) and low cost (e.g., the cost of Na_2_CO_3_ is about 25–30 times lower than that of Li_2_CO_3_) [1–3]. Since lithium and sodium are both alkali metals and have similar chemical and electrochemical properties, both academia and industry acknowledge the importance of SIBs and actively seek to use existing lithium-ion battery technology to industrialize SIBs [4, 5]. However, compared with lithium ions (0.76 Å), the larger ionic radius of sodium ions (1.02 Å) will cause various structural evolutions during the Na^+^ insertion/extraction process, leading to problems such as deterioration of the host crystal structure and poor cycling stability [6]. At the same time, the larger atomic weight of Na (23 g mol^−1^) compared to Li (6.9 g mol^−1^), along with the higher standard electrochemical potential of Na (2.71 V for Na^+^/Na vs. 3.04 V for Li^+^/Li), poses challenges to SIBs in achieving comparable energy density to lithium-ion batteries [7]. Layered oxide cathode materials have been widely studied due to their high energy density and facile large-scale preparation [8–10]. Compared with most P2-Na_x_TMO_2_ cathodes (x varies between 0.6 and 0.8), the initial structure of O3-type Na_x_TMO_2_ (x is usually 1) is very similar to LiCoO_2_, and it can provide more capacity in the same voltage range [11–13]. Despite the high capacity, these materials exhibit complex phase transformations during the Na^+^ deintercalation process, leading to rapid capacity fading [14, 15]. Simultaneously, the cathode undergoes surface remodeling or reduction of the transition metal oxidation state in contact with the electrolyte, which indicates a charge transfer between the positive electrode and the electrolyte, a phenomenon that is more intense at high voltage [16–18]. An unstable cathode–electrolyte interphase (CEI) can lead to continuous electrolyte decomposition and transition metal dissolution during cycling [19, 20]. Therefore, it is important to implement strategies such as optimizing the electrolyte and engineering the electrode interphase to promote the development of stable and high-energy SIBs [21].

The construction of thin and dense, especially inorganic-rich CEIs with weak bonding to the cathode is essential for the effective suppression of persistent adverse reactions at the interphase, inhibition of cathode structural damage and electrolyte penetration, thus enhancing the cathode cycle life at high voltage [22, 23]. However, traditional carbonate electrolyte has low oxidation stability and strong solvation effect on CEI. Therefore, it has poor passivation ability for the charged positive electrode at > 4 V (vs. Na^+^/Na) and cannot form a stable CEI [24, 25]. For this reason, a great deal of effort has been invested in exploring high-voltage electrolytes [26, 27]. Currently effective strategies for configuring high-voltage batteries include the use of high-concentration electrolyte (HCE) and localized high-concentration electrolyte (LHCE) [28, 29]. For example, a HCE formulation consisting of 5 M sodium bis(fluorosulfonyl)imide (NaFSI) in 1,2-dimethoxyethane (DME) was studied for high-voltage applications [30]. Compared with 1 M NaFSI/DME, HCE exhibits an electrochemical window up to 5 V due to less free solvent and the presence of anions in the solvation sheath. However, HCE has shortcomings such as extremely high cost, increased viscosity and insufficient wetting ability, which hinder further applications. These effects are mitigated by incorporating non-solvent diluents into the HCE to form LHCE [31]. For example, LHCE composed of NaFSI/DME/1,1,2,2-tetrafluoroethyl-2,2,3,3-tetrafluoropropyl ether (TTE) (molar ratio of 1.0:1.2:1.0) minimizes TM dissolution when used in O3-type cathodes, promotes the formation of a stable interphase and enhances cycling stability [32]. Similarly, LHCE consisting of NaFSI/DME/1H, 1H, 5H-octafluoropentyl-1, 1, 2, 2-tetrafluoroethyl ether (OTE) in a 1.0:1.5:3.0 mol ratio allows more anions to enter the inner solvation sheath layer and facilitated the gradual decomposition of anions [33]. However, it is worth noting that many LHCEs for SIBs are still mainly composed of ether-based solvents, which brings practical challenges to oxidation stability for high-voltage applications on the cathode side [34, 35]. In contrast, sulfone solvents with high oxidation resistance, high dielectric constant and low flammability appear to be more promising for high-voltage applications [36]. However, sulfone-based electrolytes have received limited attention due to the fact that they also have the disadvantages of high melting point, high viscosity and poor wettability to electrodes and diaphragms [37]. To date, stable and efficient cycling in SIBs based on sulfone electrolytes has not been reported. The development of LHCEs with excellent oxidation resistance and the construction of homogeneous and dense CEIs are essential for enhancing the commercial viability of SIBs and achieving higher energy density.

In this work, LHCE of 1.2 mol L^−1^ NaTFSI/SUL:OTE:FEC was prepared using NaTFSI, sulfolane (SUL) and diluent OTE (SUL and OTE molar ratio of 1:1) with 5 wt% of FEC as additive. A comprehensive study was conducted to elucidate the relationship between the solvated structure of the electrolyte and the layered oxide cathode interphase. NaTFSI/SUL:OTE:FEC has a higher electrochemical stabilization window compared to commercial carbonate electrolyte (E-Control). And it decomposes into homogeneous and dense S, N-rich inorganic interphases during the cycling process, which gives it good compatibility with NaNi_1/3_Mn_1/3_Fe_1/3_O_2_ cathode. The cell based on NaTFSI/SUL:OTE:FEC has a high reversible capacity of 130.82 mAh g^−1^ at 1 C in the voltage range of 2–4.2 V. After undergoing 300 cycles, it demonstrates an impressive 79.48% capacity retention (34.27% for E-Control), and its nonflammable nature effectively improves the safety of SIBs.

## Experimental Section

Conductive carbon black (Super P) and poly(vinylidene difluoride) (PVDF) were bought from the Hefei Kejing Material Technology Co., Ltd. The N-methyl-2-pyrrolidone (NMP), sulfolane (SUL), 1H, 1H, 5H-octafluoropentyl-1, 1, 2, 2-tetrafluoroethyl ether (OTE) and fluoroethylene carbonate (FEC) were purchased from Shanghai Aladdin Biochemical Technology Co., Ltd. Commercial carbonate electrolyte (E-Control) 1 M NaClO_4_ in DMC:EC = 1:1 vol% with 5 wt% FEC (NaClO_4_/DMC:EC:FEC) and battery-grade sodium bis(trifluoromethanesulfonyl)imide (NaTFSI) were ordered from DodoChem.

### Preparation of NaNMF Cathode Materials

NaNi_1/3_Mn_1/3_Fe_1/3_O_2_ (NaNMF) was syntheses sized through a conventional solid-state reaction. A chemometric mixture of Na_2_CO_3_ (99.9%; Aladdin), NiO (99%; Aladdin), Mn_2_O_3_ (98%; Aladdin) and Fe_2_O_3_ (99%; Aladdin) was milled in a mortar at stoichiometric ratio by planetary ball-milling with acetone at 300 rpm for 10 h by using a planetary ball mill (MITR ZQM-2L/4L). The mixture was dried and pressed into pellets, followed by heating for 14 h in air at 950 °C. At the end of the calcination, when the temperature dropped to approximately 100 °C, the samples were removed from the furnace and immediately transferred to an argon-filled glove box. The samples were cooled to room temperature in the glove box and were kept inside to avoid the exposure to moisture in air.

### Preparation of Electrolyte

All operations are conducted under an argon-filled glove box (O_2_/H_2_O < 0.1 ppm). NaTFSI was dried at 90 °C overnight in an argon-filled glovebox before use, and all the solvents were treated with 4 Å molecular sieves. Then NaTFSI was dissolved in various solvents to prepare the electrolytes. Detailed compositions of the electrolytes are provided in Table S1.

### Preparation of NaNMF Electrode

The NaNMF powder, Super P and PVDF with a weight ratio of 85:8:7 were uniformly blend in NMP solvent to prepare electrode slurry; then, the slurry was coated onto an aluminum current collector. The prepared electrode was dried in the vacuum at 110 °C for 12 h and punched into small disks with a diameter of 13 mm.

### Characterization

The morphologies and microstructures of the samples were characterized by scanning electron microscopy (SEM, JSM 7800F, JEOL) and transmission electron microscopy (TEM, JEOL JEM-F200). The chemical components of the samples were determined by X-ray photoelectron spectroscopy (XPS, K-Alpha+, Thermo Fisher Scientific). Raman spectra were recorded by using a Renishaw inVia Raman microscope with λ = 532 nm laser radiation. The crystallographic information of the synthesized products was examined by X-ray diffraction (XRD) employing Rigaku Miniflex II XRD instrument and filtered Cu Kα radiation (λ = 1.5418 Å). Standard examinations were executed with a scanning velocity of 5° per minute, spanning an angular range from 5° to 90°. The crystal structure information of the NaNMF charging process is carefully collected using specially designed in situ mold cell. The molded cell is equipped with holes for the light beam to pass through for the construction of the in situ cell. After the cell is assembled, the opening of the molded battery top housing is securely sealed with Kapton tape. Before starting in situ XRD, the electrodes with a load mass of 11 mg cm^−2^ were left to stand in the in situ cell for 3 h. Throughout the in situ experiment, the battery was charged at a steady rate of 24 mA g^−1^ over a voltage range of 2–4.2 V using the LANHE battery tester. 3D reconstruction images were obtained by using PHI nano TOF II time-of-flight secondary ion mass spectrometry (TOF–SIMS). The dissolved Ni was determined by an inductively coupled plasma optical emission spectrometer (ICP-OES, Agilent 5110). In situ electrochemical impedance spectroscopy (In situ EIS) tests were performed on an electrochemical workstation (Solartron, USA) and 2032-coin cells. The charging and discharging voltages were in the range of 2–4.2 V with the 0.2 C rate. The EIS tests were performed in the frequency range of 1,000,000–0.1 Hz with an amplitude of 10 mV. The cells were run for different cycles at a constant current density prior to the in situ EIS test. The DRT transformations were performed using the software MATLAB, and all the parameters were kept consistent throughout the calculations to ensure consistency of the DRT results. The viscosity of the electrolytes was determined on a MSK-SFM-VT Viscometer at 28 °C. ^23^Na-nuclear magnetic resonance (NMR) analysis of the electrolytes was performed using a Jeol ECZ500R 500 MHz NMR spectrometer. Prior to the test, dimethyl sulfoxide (DMSO-d6), as a deuterium reagent, was thoroughly mixed with the electrolyte.

### Electrochemical Methods

The CR2032-coin cells were assembled with NaNMF as cathode and Na metal as anode. Whatman glass fiber/D was used as the separator (19 mm diameter). The amount (150 µL) of electrolyte was used in each cell. The half-cells were tested at 0.5 or 1 C in the voltage range of 2–4, 2–4.1 or 2–4.2 V. The loading of NaNMF is around 3.3 mg cm^−2^ for half-cells. LAND CT2001A test system and Neware (CT-4008) were used to record the electrochemical performances. Here, a 1 C rate corresponds to a current density of 120 mA g^−1^. For the test of SEI dissolution, Na||Cu cells were assembled, in which Cu (19 mm diameter) is the working electrode, and Na (15.6 mm diameter) is the counter electrode. LSV curves were obtained using stainless steel as the working electrode, and Na foils as the counter and reference electrodes for positive and negative scan in a CHI660E electrochemical work station. The scanning rate was 0.1 mV s^−1^, and the scanning voltage ranges are open-circuit voltage (OCV) − 5.5 V. To evaluate the overpotential in Na||NaNMF cells after the specified cycle number, galvanostatic intermittent titration technique (GITT) was conducted with current pulse intervals at 0.1 C for 25 min, followed by 120-min rest within a voltage range of 2–4.2 V. The ionic conductivities of electrolytes were measured through assembling symmetric stainless steel cells (Fig. S1). The 1 M NaPF_6_ in DEGDME was used as the conductivity standard solution for calibration (7.476 mS cm^−1^ at 25 °C). The electrolytic conductivity value was obtained from the impedance spectroscopy of the customized two-electrode cell by the following equation:

$$\delta = L/A \times R$$

where *R* is the ohmic resistance, *A* is the area and *L* is the space between two stainless steel electrodes, respectively.

The Na^+^ transference number of electrolyte was determined by the potentiostatic polarization technique using an electrochemical workstation (CHI604E, China). A voltage of 10 mV was applied to a Na||Na cell for 2 h to measure the initial current *I*_*0*_, which is derived from both cation and anion transfer in the electrolyte. The steady-state current *I*_*SS*_ is solely attributed to the transportation of Na^+^ ions. EIS measurements were taken before and after the polarization step to obtain the impedance of the cells. The transference number was calculated by the following equation:

$$t_{{Na^{ + } }} = \frac{{I_{SS} \left( {\Delta V - I_{0} R_{0} } \right)}}{{I_{0} \left( {\Delta V - I_{SS} R_{SS} } \right)}}$$

where *ΔV* is the applied bias voltage, *R*_*0*_ is the initial impedance and *R*_*SS*_ is the steady-state impedance.

### Theoretical Calculations

The AIMD simulations were performed using the Vienna ab initio Simulation Package (VASP.5.4.4), employing the projector-augmented wave (PAW) formalism [38]. The Perdew–Burke–Ernzerhof (PBE) functional was employed to describe the exchange correlation energy [39]. The long-range van der Waals (vdW) dispersion interactions were accounted for using the DFT-D3 method developed by Grimme and co-workers [40, 41]. The plane-wave energy cutoff was set as 400 eV, and the Brillouin zone was sampled at the Γ point only.

The cluster calculations were carried out using Gaussian 16 package [42], with the starting structures obtained from the AIMD calculations. The Becke three-parameter hybrid method combined with the Lee–Yang–Parr correlation functional (B3LYP) [43] at the 6–311 + G (d, p) level was used to optimize geometries.

## Results and Discussions

### Physicochemical Properties of Electrolyte

**Fig. 1:**
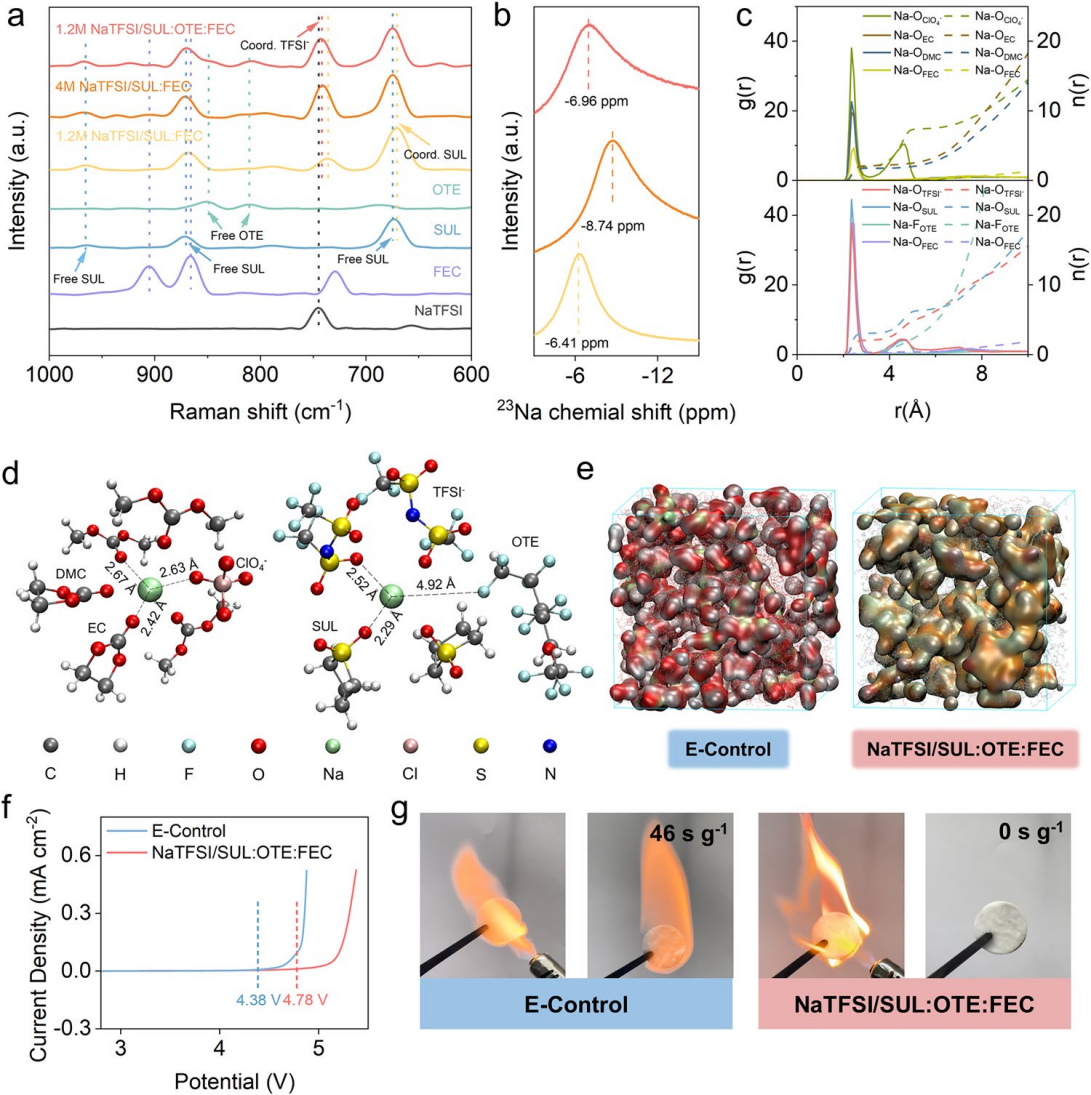
**a** Raman spectra of the different electrolytes in a wave number range from 600 to 1000 cm^−1^. **b** ^23^Na NMR spectra showing the varied Na^+^ coordination environments in different electrolytes. **c** Radial distribution functions (solid lines) and coordination numbers (dotted lines) calculated from MD simulations in E-Control and NaTFSI/SUL:OTE:FEC. **d** Coordination from MD simulations in E-Control and NaTFSI/SUL:OTE:FEC. **e** Snapshots of solvation structure of E-Control and NaTFSI/SUL:OTE:FEC. **f** LSV curves of the Na||steel half-cells with different electrolytes. **g** Flame test of E-Control and NaTFSI/SUL:OTE:FEC

Raman spectroscopy was used to explore the coordination behavior of Na^+^ and each component. As shown in Fig. 1a, the vibration peak range of TFSI^−^ anions is 740–744 cm^−1^, of which 740 cm^−1^ corresponds to free TFSI^−^ anions; 674, 871 and 966 cm^−1^ correspond to free SUL; 811 and 850 cm^−1^ correspond to free OTE [44–46]. When the NaTFSI concentration in the electrolyte increases from 1.2 to 4 M, the peak position redshifts from 740 to 744 cm^−1^, corresponding to the coordinated TFSI^−^ anion (Coord. TFSI^−^). At the same time, the redshifted peaks of SUL are 674 and 871 cm^−1^, indicating that as the concentration increases, part of the coordinated SUL is replaced by TFSI^−^. Although the NaTFSI concentration in NaTFSI/SUL:OTE:FEC is only 1.2 M, its TFSI^−^ peak and free SUL peak are consistent with those in the 4 M electrolyte. Moreover, the 811 and 850 cm^−1^ peaks corresponding to OTE have no obvious displacement, indicating that OTE is in a free state in the electrolyte and does not participate in the solvation structure.

The ^23^Na NMR spectroscopy revealed the strength of interaction between Na^+^ and TFSI^−^ in the electrolyte (Fig. 1b). The observed significant upfield (more negative) shift of 4 M NaTFSI/SUL:FEC can be attributed to the shielding effect of Na^+^, which arises due to the proximity of Na^+^ to TFSI^−^ [47]. And with the addition of OTE, that displacement is shifted slightly downfield. The shielding effect of 4 M NaTFSI/SUL:FEC is much stronger because all TFSI^−^ are well dispersed in the solvent and almost every Na^+^ is shielded and surrounded by multiple TFSI^−^. However, in 1.2 M NaTFSI/SUL:OTE:FEC, the diluent molecules separated the contact ion pairs (CIPs) and aggregates (AGGs) in the electrolyte so that the TFSI^−^ from different CIPs and AGGs could not interfere with each other, which weakened the shielding effect against Na^+^ [48]. Compared to 1.2 M NaTFSI/SUL:FEC, the 1.2 M NaTFSI/SUL:OTE:FEC peak is displaced toward the upper field, suggesting a stronger interaction of Na^+^ with TFSI^−^.

In addition, molecular dynamic (MD) simulations were performed to validate the solvated structure of NaTFSI/SUL:OTE:FEC and highlight its distinct solvation structure compared to E-Control. The radial distribution functions (RDFs) indicate that the O atoms of the solvents (DMC, EC, FEC, SUL) are more inclined to coordinate with Na^+^ to form the primary solvated shell layer (Fig. 1c). Combined with the coordination number, it is evident that both ClO_4_^−^ and TFSI^−^ are present in the inner solvated shell layer of the electrolyte. However, in the E-Control, Na^+^ exhibits a higher percentage of CIPs due to involvement of both main solvents in its coordination. In contrast, only SUL participates in solvation within the primary solvent of NaTFSI/SUL:OTE:FEC, resulting in AGGs as the predominant solvated conformation. The dominant coordination of Na^+^ with the surrounding anions or molecules in the E-Control as well as in the NaTFSI/SUL:OTE:FEC is shown in Fig. 1d. The distances between the central Na^+^ and DMC, EC and ClO_4_^−^ in E-Control are 2.67, 2.42 and 2.63 Å, respectively, indicating their presence within the primary solvated shell layer. In the case of NaTFSI/SUL:OTE:FEC, SUL and TFSI^−^ are located at distances of 2.29 and 2.52 Å from the central Na^+^, while OTE is positioned at a distance of 4.92 Å outside the internal solvation sheath. All of the above results show that TFSI^−^, SUL and FEC all successfully entered the first solvated shell layer, while OTE does not participate in the solvated structure. The MD simulation results are consistent with the results of the Raman spectra mentioned earlier. Figures 1e and S2 provide visual snapshots illustrating a more comprehensive view of the overall solvated structure for both electrolytes.

The oxidative stability of the two electrolytes was further assessed by linear scanning voltammetry at a sweep rate of 0.1 mV s^−1^ (Fig. 1f). The oxidation current density of the E-Control exhibited a rapid increase beyond 4.38 V, indicating limited oxidation stability. In contrast, NaTFSI/SUL:OTE:FEC maintained a lower current density till 4.78 V, exhibiting a broader electrochemical window. The Na^+^ transference number of NaTFSI/SUL:OTE:FEC is shown in Fig. S3. Most commercially available electrolytes suffer from high flammability that poses safety risks to batteries. To evaluate the flammability of the two electrolytes, we fully saturated GF/D (glass fibers) with each electrolyte in a volume of 300 μL and ignited them to measure self-extinguishing time [49]. As shown in Fig. 1g, the E-Control was found to catch fire easily and burn continuously with a longer self-extinguishing time (SET, 46 s g^−1^, Video S1) than NaTFSI/SUL:OTE:FEC (SET, 0 s g^−1^, Video S2). This is closely related to the low flammability of sulfolane and the high F content of NaTFSI/SUL:OTE:FEC, which is of great significance for improving the safety of electrolyte [50].

### Electrochemical Performance

**Fig. 2:**
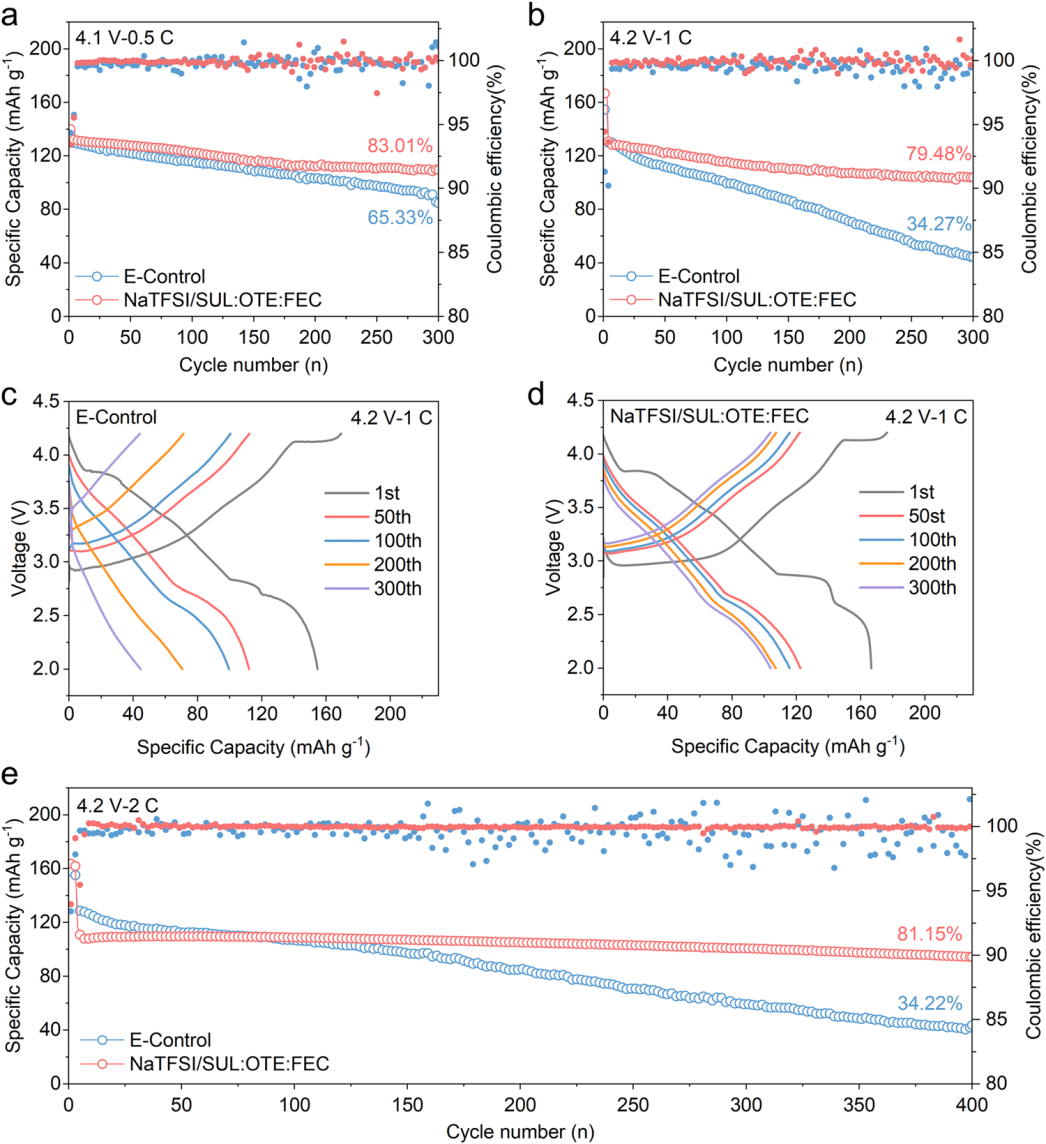
**a, b** Cycling performance of Na||NaNMF cells using E-Control and NaTFSI/SUL:OTE:FEC in the voltage range of 2–4.1 V at 0.5 C (**a**) and 2–4.2 V at 1 C (**b**). **c**, **d** Galvanostatic charge–discharge curves of Na||NaNMF cells using E-Control (**c**) and NaTFSI/SUL:OTE:FEC (**d**) in the voltage range of 2–4.2 V at 1 C. **e** Cycling performance of the Na||NaNMF cells using E-Control and NaTFSI/SUL:OTE:FEC in the voltage range of 2–4.2 V at 2 C

We utilized the most common O3-type NaNi_1/3_Mn_1/3_Fe_1/3_O_2_ (NaNMF) (Figs. S4, S5) as the cathode material for the demonstration. The electrochemical performance of NaTFSI/SUL:OTE:FEC and E-Control was tested in Na||NaNMF batteries. Initially, the O3-type NaNMF material provided a capacity close to 160 mAh g^−1^ at 0.1 C (1 C = 120 mA g^−1^), with significant plateau voltages occurring at 3 and 4.15 V (Fig. S6). Both electrolytes exhibited similar capacity retention rates after 300 cycles in the voltage range of 2–4 V, at 87.92% and 85.20%, respectively (three cycles of 0.1 C activation) (Fig. S7). Moreover, under the voltage range of 2–4.1 V and 0.5 C, NaTFSI/SUL:OTE:FEC presents 83.01% capacity retention after 300 cycles, surpassing the E-Control's 65.33% stability (Fig. 2a). Expanding to 2–4.2 V at 1 C, NaTFSI/SUL:OTE:FEC has a capacity retention rate of 79.48% after 300 cycles, while the E-Control is only 34.27% (Fig. 2b). At the same time, by comparing the cycling performance of 1.2 M NaTFSI/SUL:FEC and 4 M NaTFSI/SUL:FEC in the voltage range of 2–4.2 V (Fig. S8), it can be found that in 1.2 M NaTFSI/SUL:FEC, although SUL has good antioxidant properties and is relatively stable in the first 150 cycles. However, due to the presence of a large number of free anions and solvent molecules in the electrolyte, interfacial reactions continue to occur, resulting in a decrease in the cycle stability. In 4 M NaTFSI/SUL:FEC, due to its extremely high viscosity, normal interfacial transport of ions cannot be achieved, and thus, the electrochemical performance cannot be exerted. Comparison of galvanostatic charge–discharge and dQ/dV curves revealed the superior capacity retention of NaTFSI/SUL:OTE:FEC over the E-Control (Figs. 2c, d and S9). Rate capability of the cells in these two electrolytes is shown in Fig. S10. Similarly, the cell containing NaTFSI/SUL:OTE:FEC exhibits excellent cycling stability at a higher rate of 2 C, with 81.15% capacity retained over 400 cycles, which is significantly higher than that of E-Control (34.22%) (Fig. 2e). Furthermore, by observing the Coulombic efficiency (CE) of various cycles, it can be found that CE of NaTFSI/SUL:OTE:FEC exhibits greater stability compared to that of E-Control, suggesting its enhanced reversibility for Na||NaNMF battery systems.

Considering the favorable electrochemical performance exhibited by NaTFSI/SUL:OTE:FEC, it emerges as a valuable electrolyte for high-voltage SIBs. To explore different electrolyte compositions, four molar ratios of SUL:OTE = 1:2, 1:1, 1.5:1 and 2:1 were prepared with a base salt concentration of 1.2 M and compared by rate capability (Fig. S11a). It is evident that the optimal rate capability is achieved with a SUL:OTE rate of 1:1 (i.e., NaTFSI/SUL:OTE:FEC). In contrast, SUL:OTE at a ratio of 1:2 exhibits the worst rate capability. TEM analysis reveals that unlike the other three electrolytes, SUL:OTE at a ratio of 1:2 forms an inhomogeneous and unstable CEI, due to insufficient dissolution of NaTFSI by SUL. Furthermore, higher concentrations of SUL in the electrolyte (SUL:OTE ratios of 1.5:1 and 2:1) result in decreased levels of NaTFSI concentration within SUL solution, leading to reduced TFSI^−^ content within the first solvated sheath layer. Consequently, more SUL infiltrates this solvation sheath and decomposes at the cathode interphase, resulting in thicker CEIs which impede efficient Na^+^ transport at high rates (Fig. S11b–e).

Furthermore, we also explored the effect of FEC content in the NaTFSI/SUL:OTE:FEC system on the cycling performance. As shown in Fig. S12, an appropriate amount of FEC (5%) can effectively improve cycling performance of NaTFSI/SUL:OTE:FEC at high voltage. At the same time, a series of experiments were conducted on electrolytes with different NaTFSI concentrations in the SUL:OTE:FEC system. It can be seen that 1.2 M NaTFSI/SUL:OTE:FEC exhibited the best performance at high voltage (Fig. S13a) as well as moderate antioxidant potential and ionic conductivity (Fig. S13b, c and Table S1).

### Analysis of the NaNMF Cathode

**Fig. 3:**
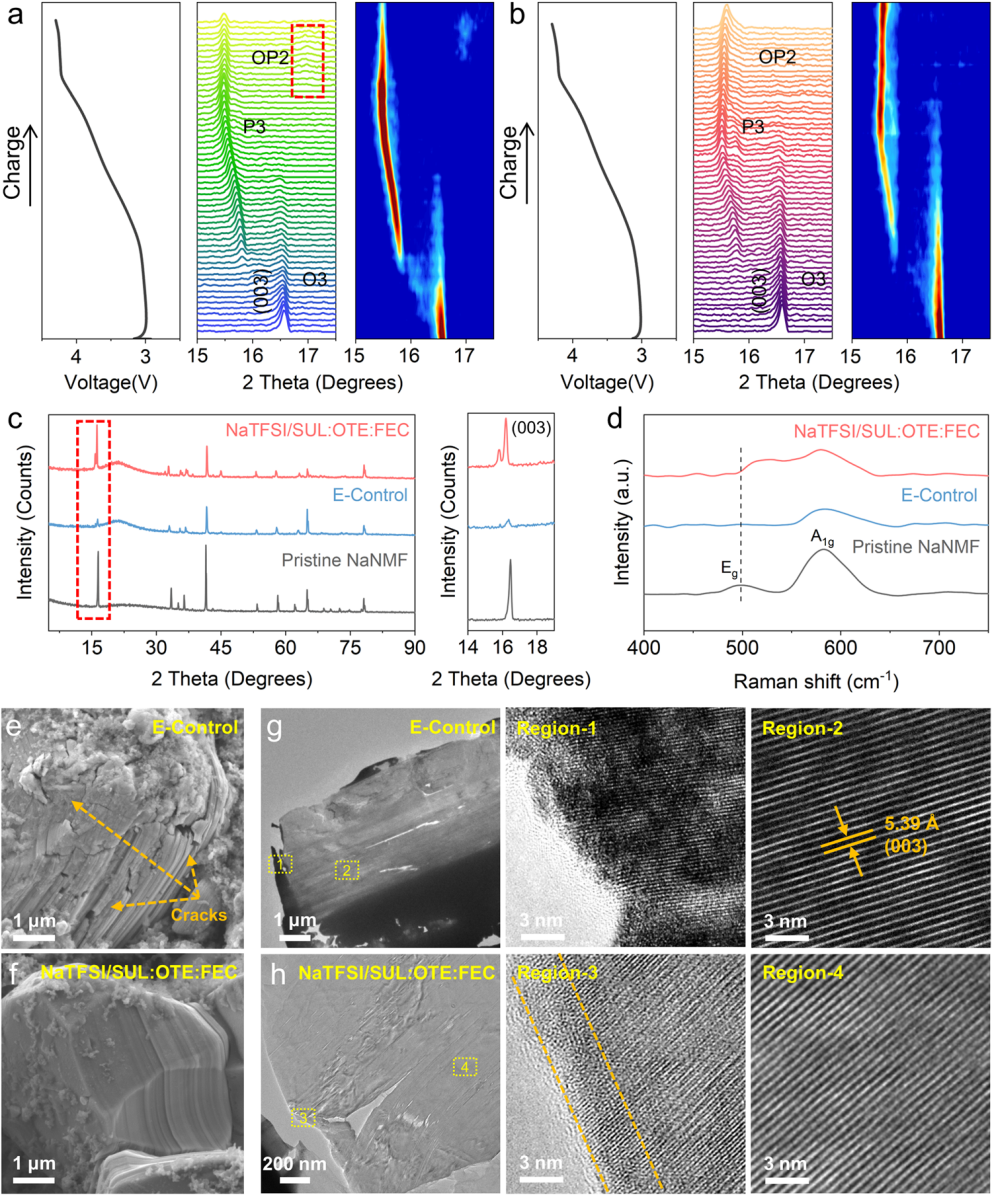
**a, b** In situ XRD testing and corresponding contour plot of NaNMF cycled in E-Control (**a**) and NaTFSI/SUL:OTE:FEC (**b**) at first charge. **c** XRD patterns and **d** Raman spectra of pristine NaNMF and after 100 cycles tested in E-Control and NaTFSI/SUL:OTE:FEC. **e**, **f** SEM images of NaNMF cathode cycled in E-Control (**e**) and NaTFSI/SUL:OTE:FEC (**f**). **g**, **h** HRTEM images of NaNMF cathode cycled in E-Control (**g**) and NaTFSI/SUL:OTE:FEC (**h**)

In situ XRD (Figs. 3a, b and S14, S15) was performed to investigate the structural stability and phase transition of NaNMF during the first charge from OCV to 4.2 V. Obviously, the intensity of (003) peak of the O3 phase is decreased as Na^+^ is shed during charging [15, 51]. With further extraction of Na^+^, the diffraction peaks of O3 completely disappear and hexagonal P3 phase diffractions emerge, suggesting an increase in oxygen electrostatic repulsion between the layers due to Na^+^ removal. This leads to an expansion of the c lattice parameter [52]. For the phase transformation of NaNMF from O3 to P3, it is evident that the E-Control has a more rapid phase transition compared to that in the NaTFSI/SUL:OTE:FEC, and rapid phase transitions often lead to abrupt changes in the cell parameters, resulting in structural damage [53]. Subsequently, upon continued charging to approximately 4.15 V, the P3 phase of NaNMF tested in the E-Control gradually diminishes and exhibits a tendency for the (003) peak to shift toward higher angles, resulting in the formation of the OP2 phase (refer to the XRD peaks marked by the red squares in Fig. 3a). The OP2 phase consists of octahedral and prismatic layers stacked alternately along the c-axis, as first reported by Yabuuchi et al. [54]. This structural transformation contributes significantly to lattice variations and volume changes that cannot be ignored. However, when tested in NaTFSI/SUL:OTE:FEC at 4.15 V, the NaNMF effectively suppresses this detrimental P3 to OP2 phase transition, thereby mitigating volumetric changes during cycling.

The cycled NaNMF cathode was systematically characterized to confirm the structural effects of the two electrolytes after undergoing high-voltage and long cycling. The (003) peak of the post-cycled NaNMF in NaTFSI/SUL:OTE:FEC, as depicted in Fig. 3c, remains well preserved. Conversely, when tested with a E-Control, the (003) peak almost completely disappears, indicating the destruction of the layered structure. In terms of the Raman spectrum (Fig. 3d), the characteristic peak around 497 cm^−1^ is the E_g_ mode, representing two-dimensional changes within the ab-plane. Additionally, the characteristic peak at around 608 cm^−1^ represents the A_1g_ mode and signifies one-dimensional changes of the c-axis [55]. After testing 100 cycles with E-Control, no E_g_ peak can be detected at the cathode, which indicates that the layered structure is destroyed. However, the E_g_ peak in NaTFSI/SUL:OTE:FEC is still detectable. This demonstrates that NaTFSI/SUL:OTE:FEC exhibits significant protective effects on maintaining structural integrity under high-voltage conditions.

Figures 3e and S16a present that NaNMF particles tested in E-Control experienced significant slippage and numerous cracks after 200 cycles, which is further supported by the cross-sectional view (Fig. S17b). These cracks are generally induced by phase transition strain during the cycling process. During the phase transition, the single crystallite c/a ratio and the boundary strain of different phases change significantly, resulting in lattice distortion at the phase boundary, which drives the formation and propagation of cracks during the sodiumization/desodiumization process [56]. In contrast, the NaNMF particles in NaTFSI/SUL:OTE:FEC exhibit an intact structure (Figs. 3f and S16b), as confirmed by the cross-sectional view revealing their structural integrity without visible cracks (Fig. S17c). In order to gain deeper insight into the underlying mechanism responsible for the exceptional cycling performance of NaTFSI/SUL:OTE:FEC at high voltage, HRTEM analysis was conducted on the cycled NaNMF after 200 cycles. As shown in Figs. 3g and S18, a large number of stacking faults and dislocations were observed on the surface area of NaNMF particle after cycled in E-Control. Conversely, the phase change region of NaTFSI/SUL:OTE:FEC appeared much thinner than that of E-Control, and it also retains most of the layered structure in the surface area (Figs. 3h and S19).

### Analysis of CEI

**Fig. 4:**
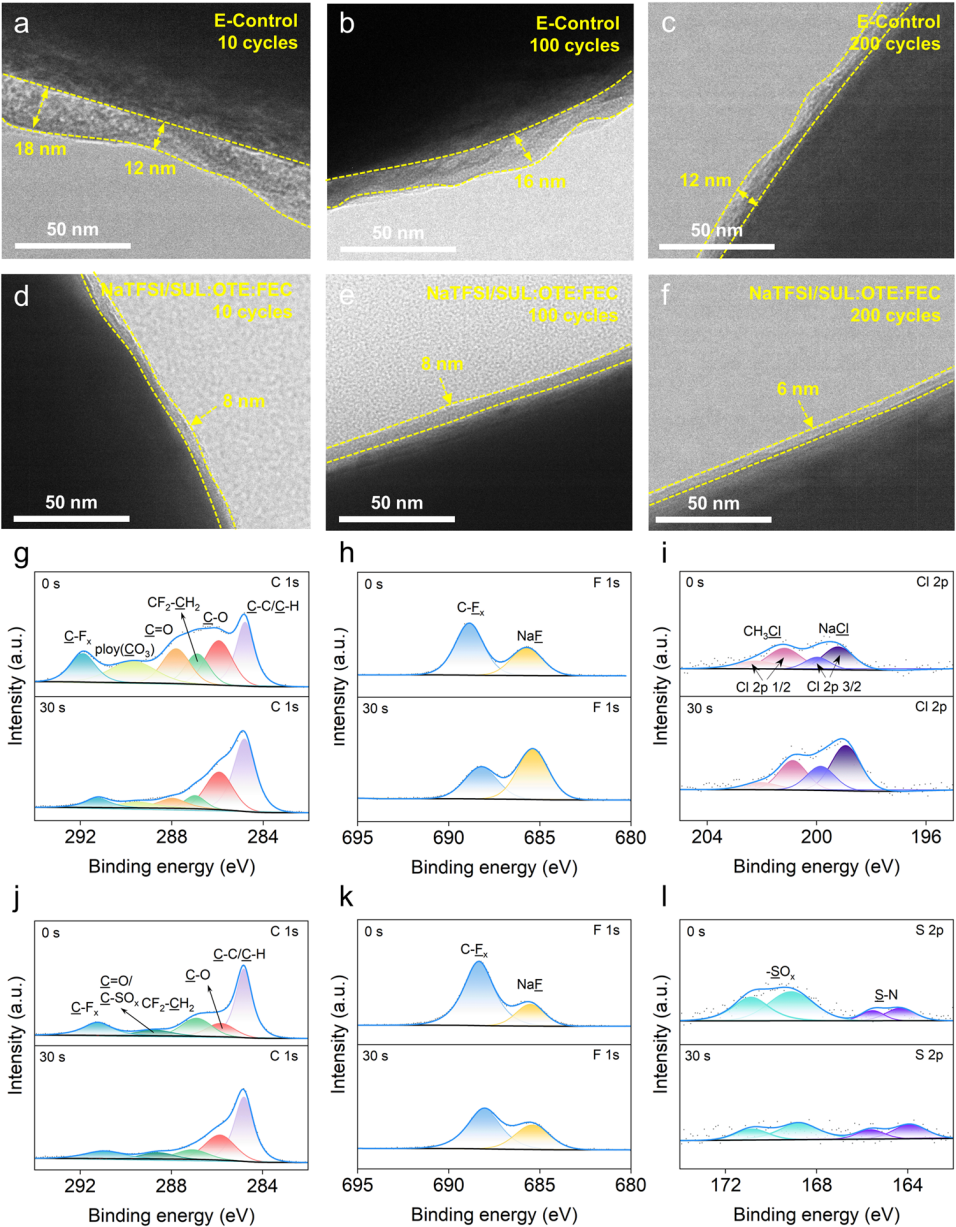
**a**–**f** TEM images of NaNMF cathode cycled in the E-Control (**a–c)** and NaTFSI/SUL:OTE:FEC (**d**–**f)**. **g** C 1*s*, **h** F 1*s* and **i** Cl 2*p* XPS spectra of the cycled NaNMF cathodes after 200 cycles in E-Control. **j** C 1*s*, **k** F 1*s* and **l** S 2*p* XPS spectra of the cycled NaNMF cathodes after 200 cycles in NaTFSI/SUL:OTE:FEC

The morphology of CEI on the NaNMF cathode surface was investigated using transmission electron microscopy (TEM). As shown in Fig. 4a–c, the CEI thickness is uneven and varies during the first 200 cycles when tested in the E-Control. In contrast, NaTFSI/SUL:OTE:FEC maintained a thin and dense CEI after different cycles (Fig. 4d–f). This thin CEI indicates reduced side reactions and minimal volume variation. Combined with the above structural characterization results, it is obvious that the thin and dense CEI effectively isolates the continuous reaction between the cathode and the electrolyte, reducing the continuous damage to the cathode interface and the further expansion of surface cracks [57, 58]. At the same time, fewer stacking faults and dislocations indicate that NaTFSI/SUL:OTE:FEC significantly contributes to the excellent cycling stability of NaNMF at high voltage.

In addition, X-ray photoelectron spectroscopy (XPS) was used to determine the composition of the CEI on the surface of the NaNMF cathode after 200 cycles (Figs. 4g–l and S20, S21). For CEI formed in E-Control, the main peaks identified in the C 1*s* spectra (Fig. 4g) were C–C/C–H, C–O, C = O and poly(CO_3_). CH_2_–CF_2_ and C–F_x_ originate from the polyvinylidene fluoride (PVDF). At the same time, it can be seen that the surface of unetched NaNMF tested in E-Control forms a CEI rich in polycarbonate, ROCOONa and Na_2_CO_3_, which are the decomposition products of carbonates (DMC, EC) under high voltage [59]. After 30 s of Ar^+^ etching to remove the effects of contaminants on the surface of the cathode, the presence of distinct C=O and poly(CO_3_) peaks was still observed, demonstrating that the CEI formed by E-Control was enriched with organic derivatives decomposed by EC and DMC. The organic CEI components and derived Na_2_CO_3_ in the electrolyte are often unstable and therefore exhibit poor high-voltage stability [60]. In contrast, the CEI in NaTFSI/SUL:OTE:FEC shows C-SO_x_ peaks decomposed by NaTFSI and SUL, and its components are similar before and after etching, indicating that the internal and external components of CEI are uniform (Fig. 4j). In the F 1*s* spectrum (Fig. 4h, k), the peak area ratio of E-Control changes significantly before and after etching. It should be noted that there is a large amount of NaF after etching for 30 s, which will be further discussed later. The larger proportion of C-F_x_ peaks in the F 1*s* spectrum of NaTFSI/SUL:OTE:FEC before etching may be due to the decomposition of free OTE [61]. The Cl 2*p* spectrum (Fig. 4i) indicates that the primary decomposition products of NaClO_4_ in E-Control are CH_3_Cl and NaCl. Furthermore, it reveals significant compositional differences inside and outside the CEI before and after etching. S 2*p* (Fig. 4l) and N 1*s* (Fig. S21) illustrate the decomposition products of NaTFSI and SUL in CEI. The presence of inorganic components rich in S and N elements on both the surface and interior of CEI in NaTFSI/SUL:OTE:FEC contributes to enhancing its physical strength.

**Fig. 5:**
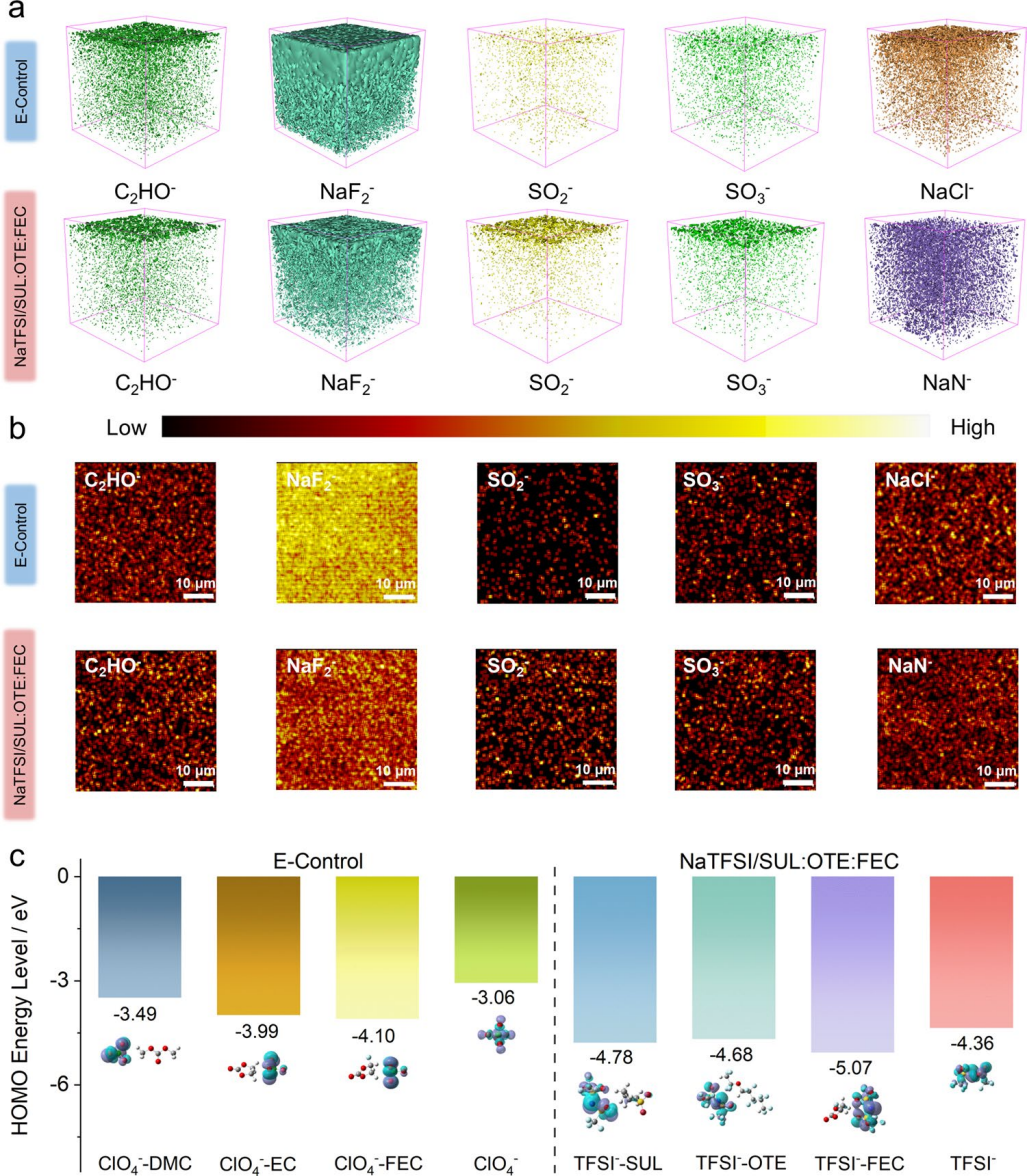
**a** TOF–SIMS 3D profiling and **b** TOF–SIMS 2D surface mappings of several secondary ion fragments C_2_HO^−^, NaF_2_^−^, SO_2_^−^, SO_3_^−^, NaCl^−^ and NaN^−^ on NaNMF cathodes after being cycled with E-Control and NaTFSI/SUL:OTE:FEC. **c** Calculated HOMO energy levels of a single anion molecule and anion-solvent in E-Control and NaTFSI/SUL:OTE:FEC

In order to further study the distribution of each CEI component on the cathode, an in-depth analysis using time-of-flight secondary ion mass spectrometry (TOF–SIMS) was performed. The TOF–SIMS depth distribution (normalized to the maximum value) of NaNMF cathodes tested in E-Control and NaTFSI/SUL:OTE:FEC for 20 cycles were obtained. The TOF–SIMS 3D images illustrate that the CEI formed in NaTFSI/SUL:OTE:FEC is more homogeneous and thinner, yet E-Control shows the presence of thick CEI that is inhomogeneous and loose (Figs. 5a and S23). It also contained NaCl^−^, a NaClO_4_ decomposition product, as well as higher amounts of organic debris such as CH^−^ and C_2_HO^−^. This is also demonstrated by the TOF–SIMS 2D mapping spectrum (Fig. 5b) and spatially resolved depth profile of the chemical composition (Fig. S22). The CH^−^, C_2_HO^−^ and NaCO_3_^−^ contents are higher in E-Control, indicating a violent decomposition of solvents, which is consistent with the XPS results. The high content of NaF_2_^−^ in E-Control perhaps stems from the ineffective passivation of the cathode surface. And excessive Na_2_CO_3_ in CEI leads to high pH near the electrode/binder interface, resulting in HF generation that deteriorates the interphase [62]. At the same time, energizing the electrolyte at high voltage causes the electrolyte to decompose to produce strong acids (HF, HCl), which continuously disrupts and reorganizes the fragile CEI. HF and HCl react with cathode dissolution products and TM ions, resulting in NaF permeation of the CEI [63, 64]. Unlike LiF, NaF has a high diffusion barrier and mechanical roughness for Na^+^, while its low flexibility cannot resist volume changes [65]. Excess NaF is even more unfavorable for the transport of Na^+^ [66].

In contrast, NaTFSI/SUL:OTE:FEC generated CEI exhibits an outer layer composed of S-containing substances (Fig. 5a), and S-containing substances can improve the stability of CEI by inhibiting the successive decomposition of its surface electrolyte [67]. It should be noted that the presence of S in E-Control is determined by the presence of trace amounts of SO_2_ and SO_3_ substances on the surface of the cycled cathode (Fig. S24). In addition to the S-containing substances, a homogeneous distribution of NaN^−^ can also be observed, which is usually more stable on the CEI and poorly soluble in the electrolyte [60]. It was also observed that the concentration of NiF_3_^−^, MnF_3_^−^ and FeF_3_^−^ substances was significantly lower at the cathode in the NaTFSI/SUL:OTE:FEC cycle compared to the E-Control (Fig. S23). The presence of NiF_3_^−^ indicates the leaching of transition metals from the cathode, which is caused by a significant side reaction between the cathode and electrolyte [68]. ICP testing of the glass fibers for Ni after 300 cycles yielded a residual Ni of 16 ppm for the E-Control and only 5 ppm for NaTFSI/SUL:OTE:FEC (Fig. S25). The smaller amount of Ni dissolution fully demonstrates the good protection of the cathode side by NaTFSI/SUL:OTE:FEC.

The HOMO energy levels of the anions and anion-solvents were calculated using density functional theory (DFT) (Fig. 5c) [69]. The lower the HOMO energy level, the better the antioxidant capacity. As presented, the anions are the first to break down in both E-Control and NaTFSI/SUL:OTE:FEC. It is observed that the HOMO energy levels of the three solvents coordinated with ClO_4_^−^ in the E-Control are higher than those of the three solvents of NaTFSI/SUL:OTE:FEC. This indicates that NaTFSI/SUL:OTE:FEC has better oxidation resistance than E-Control at high voltage, which is consistent with the LSV results. By comparing the HOMO energy levels of each of the ClO_4_^−^-solvent, it was observed that the two primary solvents, DMC and EC, in the E-Control underwent decomposition after ClO_4_^−^ to produce polycarbonate, Na_2_CO_3_, etc., with ClO_4_^−^-FEC exhibiting the highest antioxidant capacity. The NaTFSI/SUL:OTE:FEC system undergoes a sequential decomposition process. TFSI^−^ and SUL in the first solvation sheath will reach the cathode interface together with Na^+^, and the desolvated TFSI^−^ and SUL will enter the NaNMF crystals and decompose to form the initial CEI [59]. Combined with the S–N peaks in Figs. 4l and S21, as well as the similar contents of NSO_2_CF_3_^−^ and SO_2_CF_3_^−^ observed in Fig. S26, it is inferred that TFSI^−^ decomposes into NSO_2_CF_3_^−^ and SO_2_CF_3_^−^ in the first step, and then further decomposes into NaF, Na_3_N and SO_x_^−^ products [70]. Subsequently, SUL and FEC decompose to form CEI containing S, F and organic derivatives. Combining the above characterizations, it can be concluded that NaTFSI/SUL:OTE:FEC constitutes the CEI structure where the outer layer consists of organic derivatives and fluorinated components that enhance Na^+^ diffusion while ensuring robustness. With the incorporation of mechanically strong inorganic materials as pillars, each component is uniformly coordinated to establish a stable CEI structure capable of delivering long-term cycling performance at high voltage.

### Analysis of SEI and the Na Anode

The Na||Cu cells were assembled and tested within the voltage range of 0.005–1 V, aiming to monitor the deposit behavior of Na on the Cu foils. Subsequently, XPS analysis was conducted to examine the components of SEI. Figure S27a–c and d–f depicts the XPS results of Cu foil cycled for 100 cycles in E-Control and NaTFSI/SUL:OTE:FEC, respectively. It was observed that in E-Control, there were higher proportions of C = O and poly(CO_3_) peaks decomposed by DMC and EC. In contrast, the Cu foil cycled in NaTFSI/SUL:OTE:FEC exhibited the formation of a SEI containing NaF and Na_3_N, which effectively impedes the reaction between Na anode and the electrolyte. This result is consistent with the LUMO energy levels of Na^+^-anion and Na^+^-solvent calculated using DFT (Fig. S28). Moreover, by observing the cross section of the Na anode in the Na||NaNMF half-cell after 200 cycles (Fig. S27g, h), it can be found that the Na anode in the E-Control exhibits a more pronounced corrosion depth and experiences severe surface powdering. In contrast, within the NaTFSI/SUL:OTE:FEC system, the formation of an inorganic-rich SEI effectively shields and significantly mitigates corrosion on the Na anode.

To verify the stability of the SEI of NaTFSI/SUL:OTE:FEC on Na anode, we conducted further analysis on SEI dissolution by examining the capacity loss in Na||Cu cells with both electrolytes (Fig. S29). The cells underwent cycling within a voltage range of 0.005–2 V, with intermittent pauses of 50, 30, 15 or 5 h after every five cycles before resuming for the remaining cycles. Following each pause, there was an increase in capacity for the reduction reaction of the electrolyte, leading to the formation of a new SEI and compensating for any dissolved SEI during prolonged pauses (Fig. S29a) [71]. The results clearly show that NaTFSI/SUL:OTE:FEC exhibits significantly lower capacity loss compared to E-Control; specifically, during the 50-h pause period, the average capacity loss of E-Control is 9.33 μAh, which is approximately three times that of NaTFSI/SUL:OTE:FEC (only 2.4 µAh). Through the analysis of capacity loss after different pause times, the dissolution of SEI in NaTFSI/SUL:OTE:FEC was largely suppressed due to its unique solvation structure, resulting in the formation of a compact and robust SEI that aligns with the aforementioned characterization results.

### Characterizations of the Interphases

**Fig. 6:**
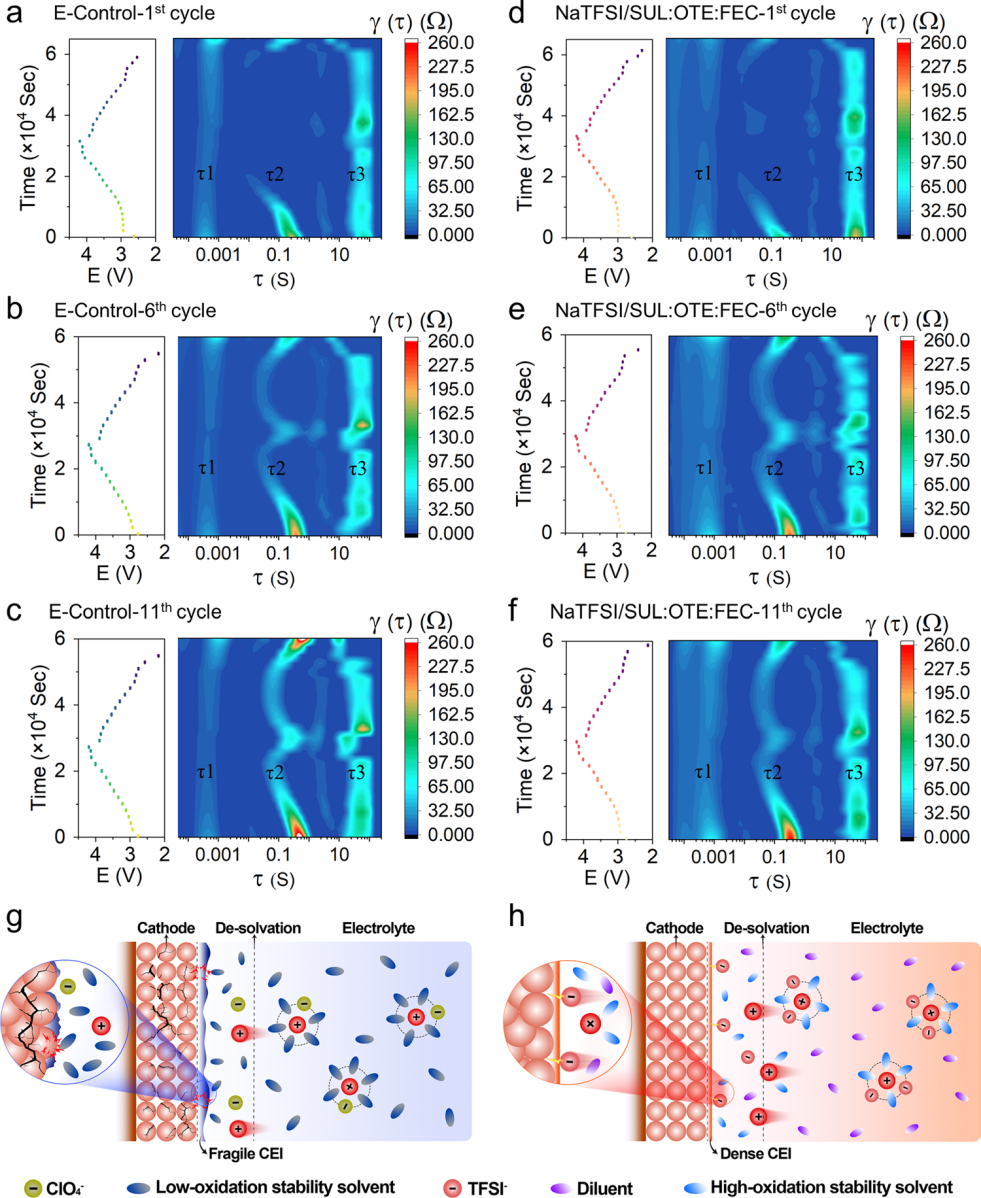
**a**–**f** In situ EIS DRT calculation and charging/discharging curves of Na||NaNMF cells for the E-Control (**a**–**c**) and NaTFSI/SUL:OTE:FEC (**d**–**f**) at the 1st, 6th and 11th cycle. **g**, **h** Schematic illustrations of the solvation structure and the CEIs in the E-Control (**g**) and NaTFSI/SUL:OTE:FEC (**h**)

To analyze the complex electrode–electrolyte interphase evolution during charging and discharging, we performed in situ EIS analyses (Figs. 6a–f and S30) and further clarified the processes through distribution of relaxation time (DRT). The mid- and low-frequency semicircles in the in situ EIS spectra exhibit dynamic evolution during charging and discharging, which can be attributed to the successive reactions of the cathode with the electrolyte and the phase transition evolution process. Three different specific relaxation times can be distinguished in the DRT results, which are, respectively, located between 10^−4^ and 10^−3^, 10^−1^ and 1, and 10 and 10^2^, and are labeled τ1, τ2 and τ3. The peak area of DRT represents the impedance value during a specific electrochemical process. High frequency τ1 and medium frequency τ2, respectively, symbolize different interphase transition responses and charge transfer evolution during the formation of CEI and SEI. The rightward shift of the τ1 peak during the charge and discharge process indicates the continuous reaction between the electrode and the electrolyte, representing the irreversible process of CEI and SEI formation [72, 73]. τ2 represents the phase evolution process on the cathode side during charge and discharge. For τ3, it represents the mass transport of the electrolyte phase in the electrolyte-filled pore [74]. Firstly, analyzed at the DRT result τ1, E-Control shows a tendency to weaken and then strengthen, indicating that a fragile CEI and SEI was formed during the cycling process and repeatedly ablated and remodeled at high voltage, while in NaTFSI/SUL:OTE:FEC, the peak of τ1 shows a trend of gradual enhancement, suggesting that uniformly dense CEI and SEI were formed during the cycling process, which effectively isolated the electrode and electrolyte and had a good protective effect on the electrode. Second at τ2, the charge transfer process shows strong reversibility in the 6th and 11th cycles. The Na^+^ concentration in the electrode or interphase affects the exchange current density, leading to a continuous change in the charge transfer impedance [72]. It can be clearly seen that the peak of E-Control at τ2 increases more and more during the cycle. In contrast, NaTFSI/SUL:OTE:FEC increased at the 6th cycle and stabilized at the 11th cycle, suggesting that the dense and homogeneous CEI and SEI contributes to the charge transfer at the interphase. After 10 cycles, the τ2 peak of NaTFSI/SUL:OTE:FEC was smaller than that of E-Control at charging to approximately 4.15 V, suggesting that the stabilized interphase made Na^+^ easier to extract. At the same time, combined with the above-mentioned in situ XRD, this may be related to the smaller degree of phase change of NaTFSI/SUL:OTE:FEC when charged to 4.15 V, which can effectively improve the stability of NaNMF at high voltage. Galvanostatic intermittent titration (GITT) and the calculated Na-ion diffusion coefficient (D_Na+_) results present remarkably higher D_Na+_ for the cathode cycled in NaTFSI/SUL:OTE:FEC (Fig. S31), confirming the accelerated interfacial diffusion kinetics and enhanced structural stability during charging and discharging process in NaTFSI/SUL:OTE:FEC [75–77].

The solvation structure of the electrolytes and the formation mechanism of CEIs are shown in Fig. 6g, h. Most of the carbonate solvents (DMC, EC) in the E-Control exist in free form and will decompose violently at high voltage due to their poor oxidation stability. This results in a large amount of organic matter and organic derivatives being distributed on the surface of CEI, which is usually manifested as an increase in CEI thickness and poor flexibility. Therefore, frequent side reactions occur at the CEI, thereby deteriorating battery performance. In contrast, in NaTFSI/SUL:OTE:FEC, the presence of OTE makes TFSI^−^ and SUL more involved in the first solvation sheath. Furthermore, due to the high oxidation resistance of SUL, the CEI is rich in inorganic substances containing S and N produced by the decomposition of TFSI^−^. The robust and dense CEI, along with enhanced oxidative stability of the electrolyte, contributes to the reduction in side reactions and effective protection of the cathode, thereby enhancing cycling stability of high-voltage SIBs.

## Conclusions

In summary, a localized high-concentration electrolyte, NaTFSI/SUL:OTE:FEC, was developed for high-voltage SIBs. The designed electrolyte forms a highly stable and robust CEI enriched with S and N species, which provides effective protection to the O3-type layered oxide NaNMF cathode during high-voltage testing at 4.2 V. Additionally, it efficiently suppresses electrolyte decomposition, transition metal dissolution and structural reconstruction of the layered oxide material. Moreover, the nonflammable nature enhances the safety of SIBs. By utilizing the sulfolane-based electrolyte, the Na||NaNMF cell exhibits an impressive reversible capacity of 130.82 mAh g^−1^ at 1 C while maintaining an impressive capacity retention rate of 79.48% after 300 cycles. Furthermore, even under high discharge conditions at 2 C, the capacity retention remains exceptionally high at 81.15% after enduring 400 cycles. This study strategically employs sulfolane, a highly oxidation-resistant compound, to enhance the stability of O3-type layered oxide cathodes when subjected to elevated voltage by optimizing both the solvation structure of Na^+^ and the interphase between the cathode and electrolyte. Additionally, it offers valuable insights into harnessing sulfolane-based electrolytes in SIBs.

## Supplementary Information

Below is the link to the electronic supplementary material.

Supplementary file1 (DOCX 16682 kb)

Supplementary file2 (MP4 3104 kb)

Supplementary file3 (MP4 429 kb)
