# Supplementary material for: Sulfolane-Based Flame-Retardant Electrolyte for High-Voltage Sodium-Ion Batteries
Source: Nanomicro Lett. 2024 Oct 18;17:45. doi: 10.1007/s40820-024-01546-7 (PMC11489388; doi:10.1007/s40820-024-01546-7)
Supplement: Supplementary file 1 — Supplementary file1 (DOCX 16682 kb) [file 40820_2024_1546_MOESM1_ESM.docx]

Supporting Information for

**Sulfolane-Based Flame-Retardant Electrolyte for High-Voltage Sodium-Ion Batteries**

Xuanlong He^1^, Jie Peng^1^, Qingyun Lin^2^, Meng Li^3^, Weibin Chen^1^, Pei Liu^1^, Tao Huang^4^, Zhencheng Huang^1^, Yuying Liu^1^, Jiaojiao Deng^1^, Shenghua Ye^1^, Xuming Yang^1^, Xiangzhong Ren^1^, Xiaoping Ouyang^1, 5^, Jianhong Liu^1, 6^, Biwei Xiao^3,^ *, Jiangtao Hu^1,^ *, Qianling Zhang^1,^ *

^1^ Graphene Composite Research Center, College of Chemistry and Environmental Engineering, Shenzhen University, Shenzhen, 518060, P. R. China

^2^ Center of Electron Microscopy, State Key Laboratory of Silicon and Advanced Semiconductor Materials, School of Materials Science and Engineering, Zhejiang University, Hangzhou, 310027, P. R. China

^3^ GRINM (Guangdong) Research Institute for Advanced Materials and Technology, Foshan, Guangdong, 528051, P. R. China

^4^ College of Energy Engineering, Zhejiang University, Hangzhou, Zhejiang 310027, P. R. China

^5^ School of Materials Science and Engineering, Xiangtan University, Xiangtan 411105, P. R. China

^6^ Shenzhen Eigen-Equation Graphene Technology Co. Ltd, Shenzhen, 518000, P. R. China

*Corresponding authors. E-mail: [zhql@szu.edu.cn](mailto:zhql@szu.edu.cn) (Qianling Zhang); [hujt@szu.edu.cn](mailto:hujt@szu.edu.cn) (Jiangtao Hu); [xiaobiwei@grinm.com](mailto:xiaobiwei@grinm.com) (Biwei Xiao)

**Supplementary Figures**


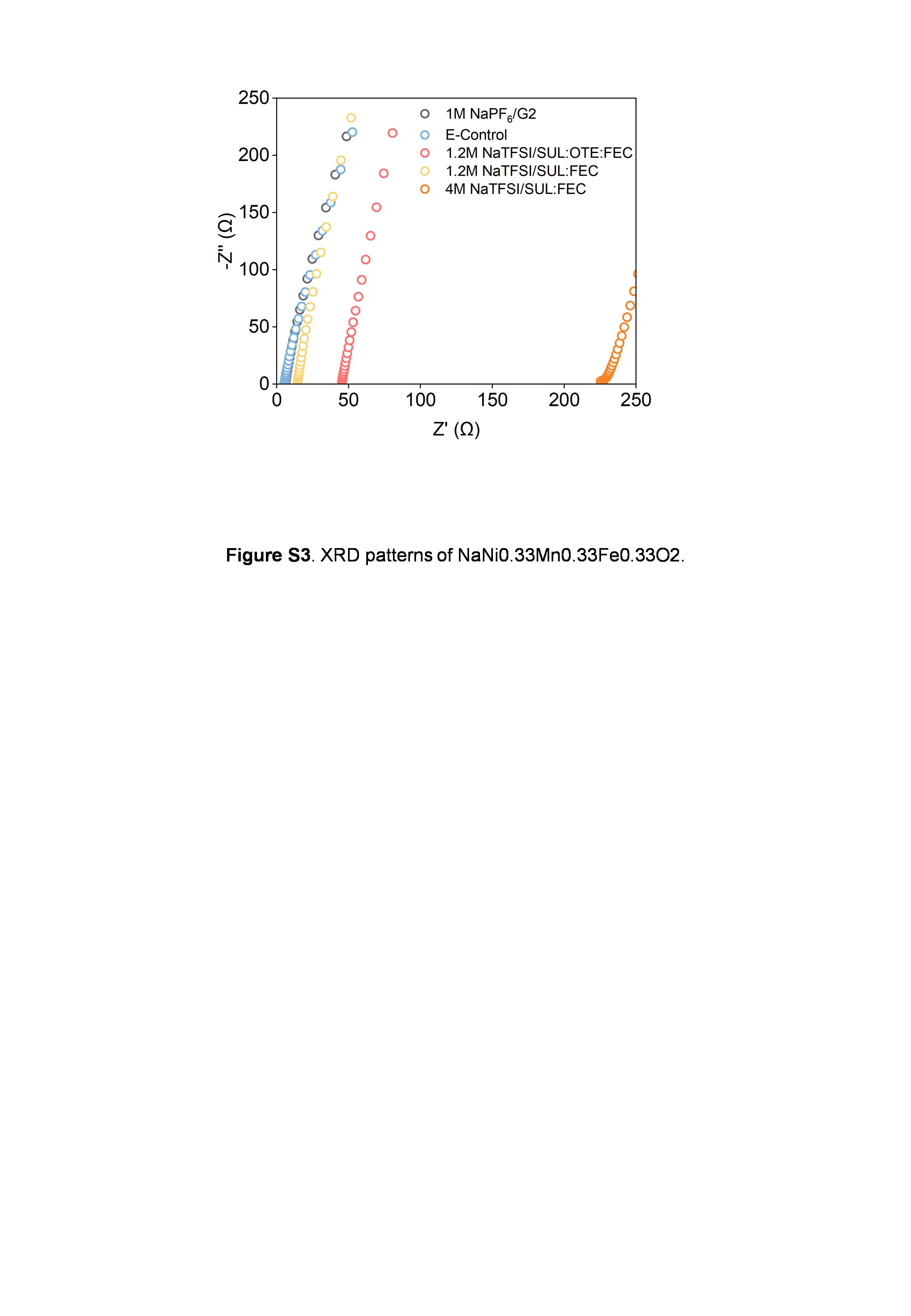


**Fig. S1** Nyquist plots of symmetric stainless steel cells assembled with different electrolytes


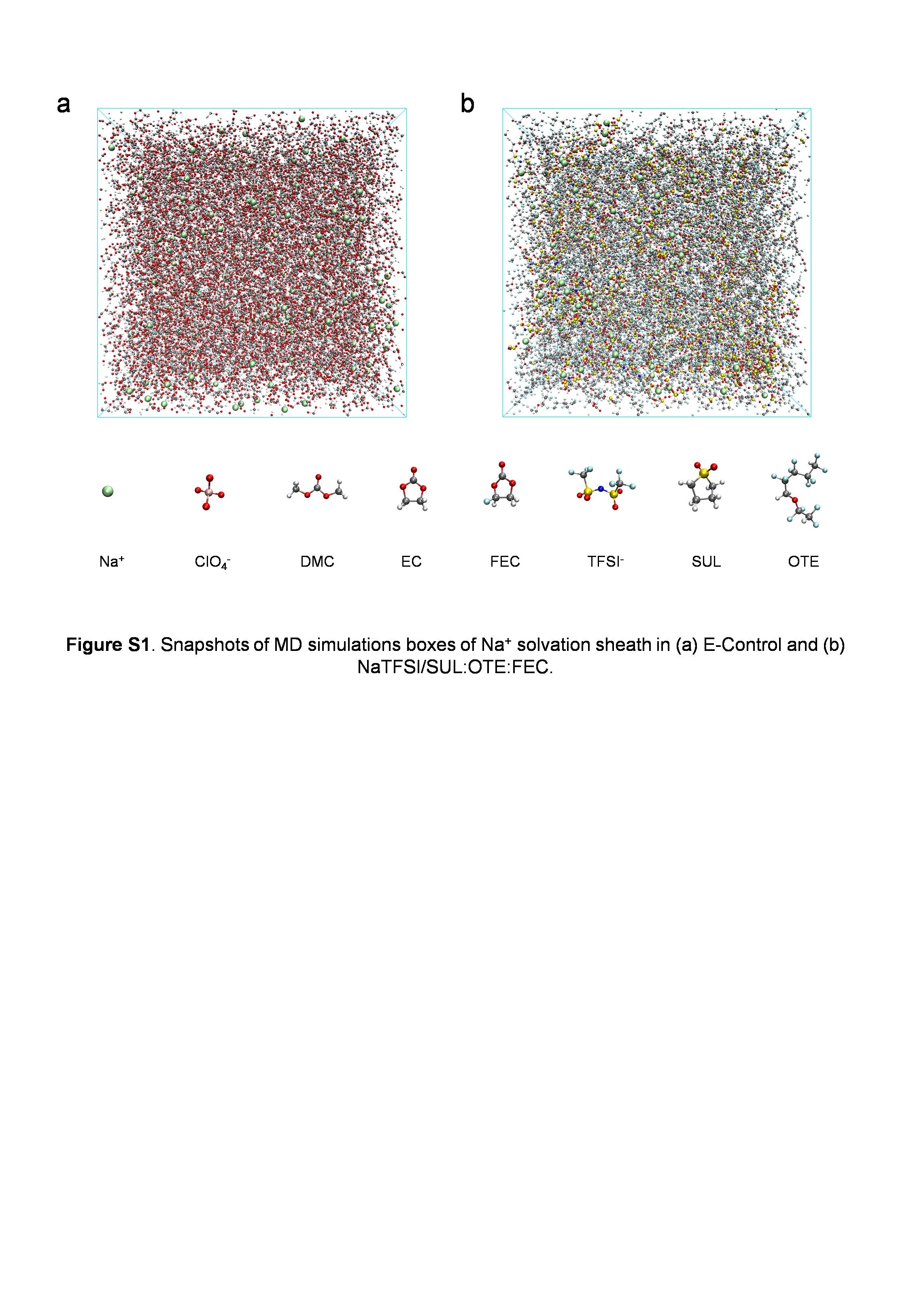


**Fig. S2** Snapshots of MD simulations boxes of Na^+^ solvation sheath in (**a**) E-Control and (**b**) NaTFSI/SUL:OTE:FEC


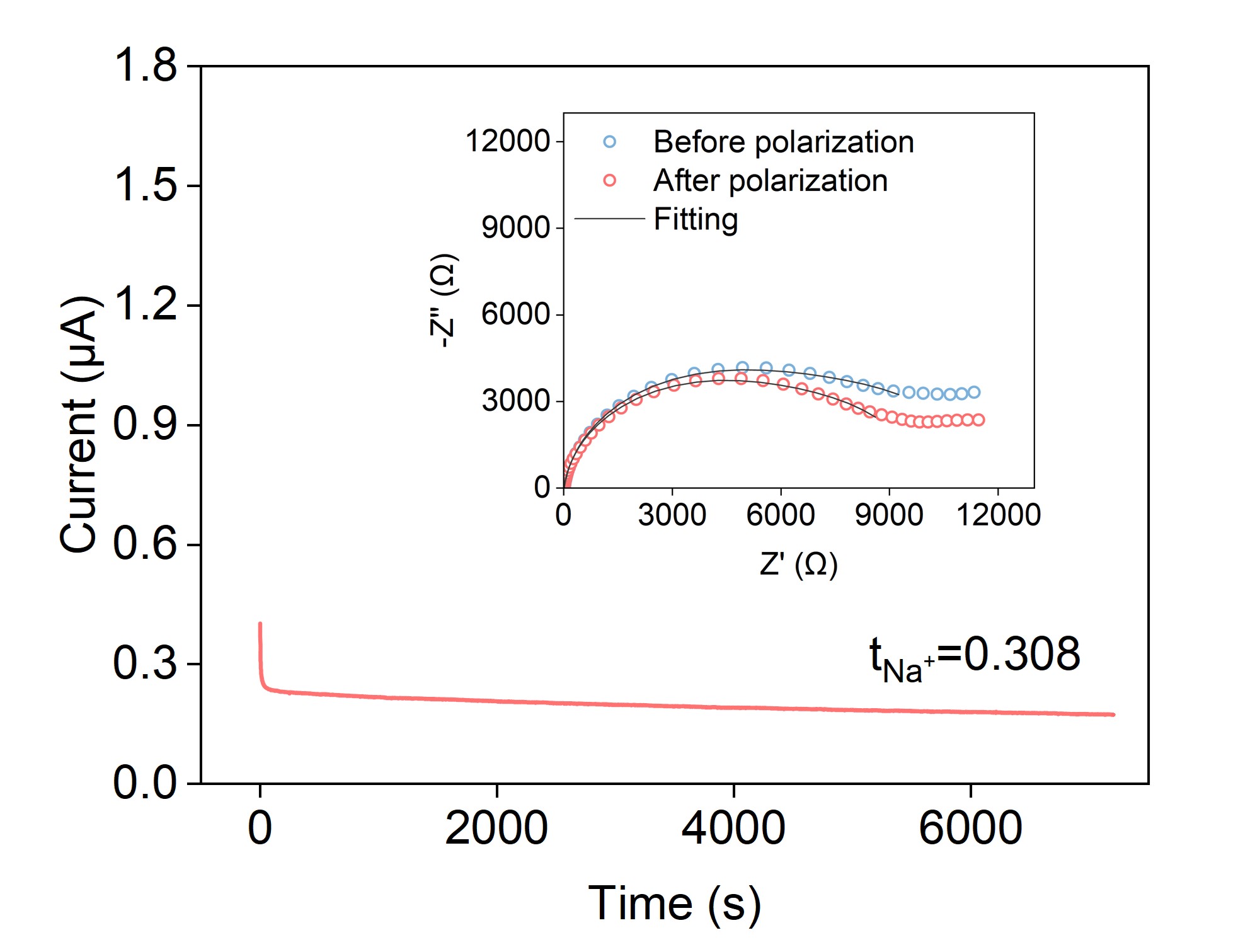


**Fig. S3** Polarization curve for NaTFSI/SUL:OTE:FEC. Inset is Nyquist impedance plots before and after polarization

**
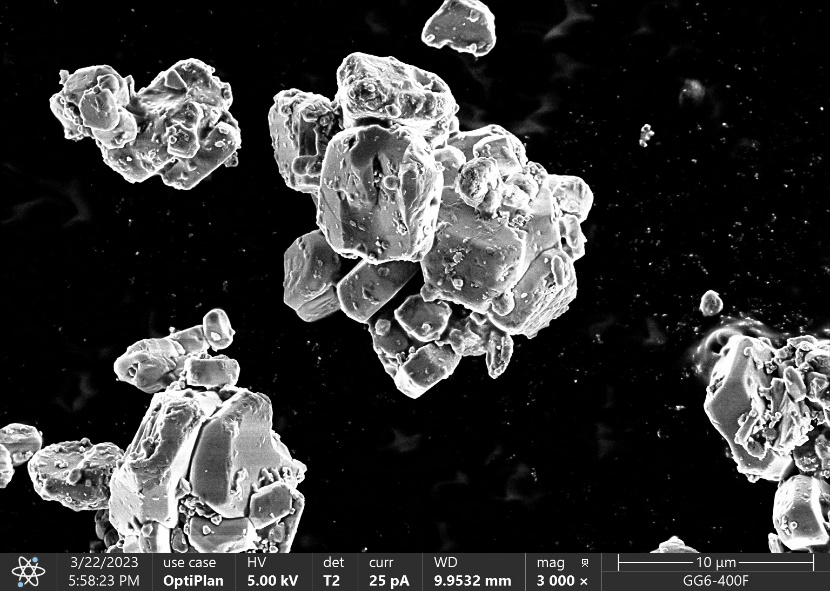
**

**Fig. S4** SEM image of NaNi_1/3_Mn_1/3_Fe_1/3_O_2_


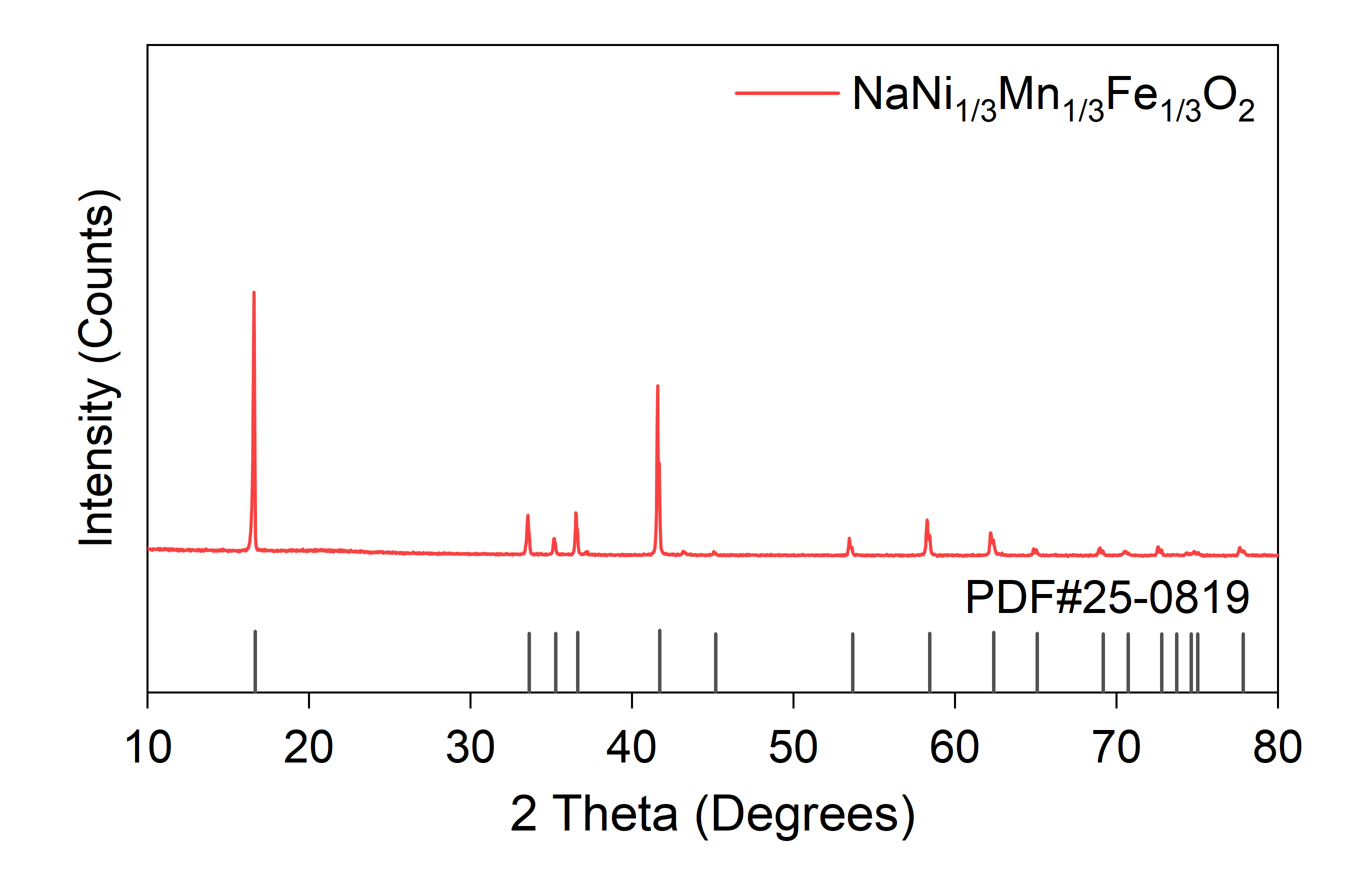


**Fig. S5** XRD pattern of NaNi_1/3_Mn_1/3_Fe_1/3_O_2_

**
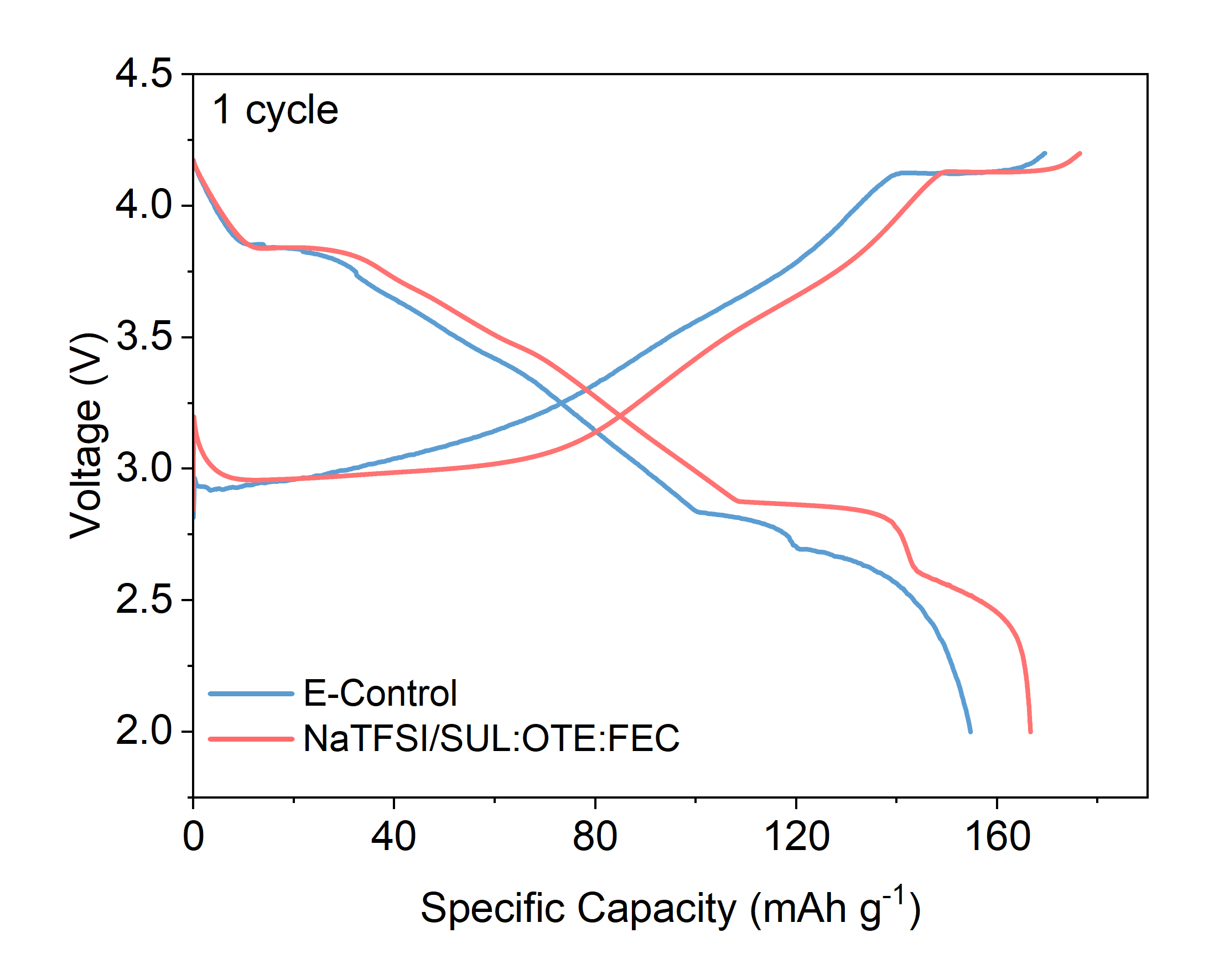
**

**Fig. S6** Initial charge/discharge curves of NaNMF cathode in different electrolytes in the voltage range of 2–4.2 V


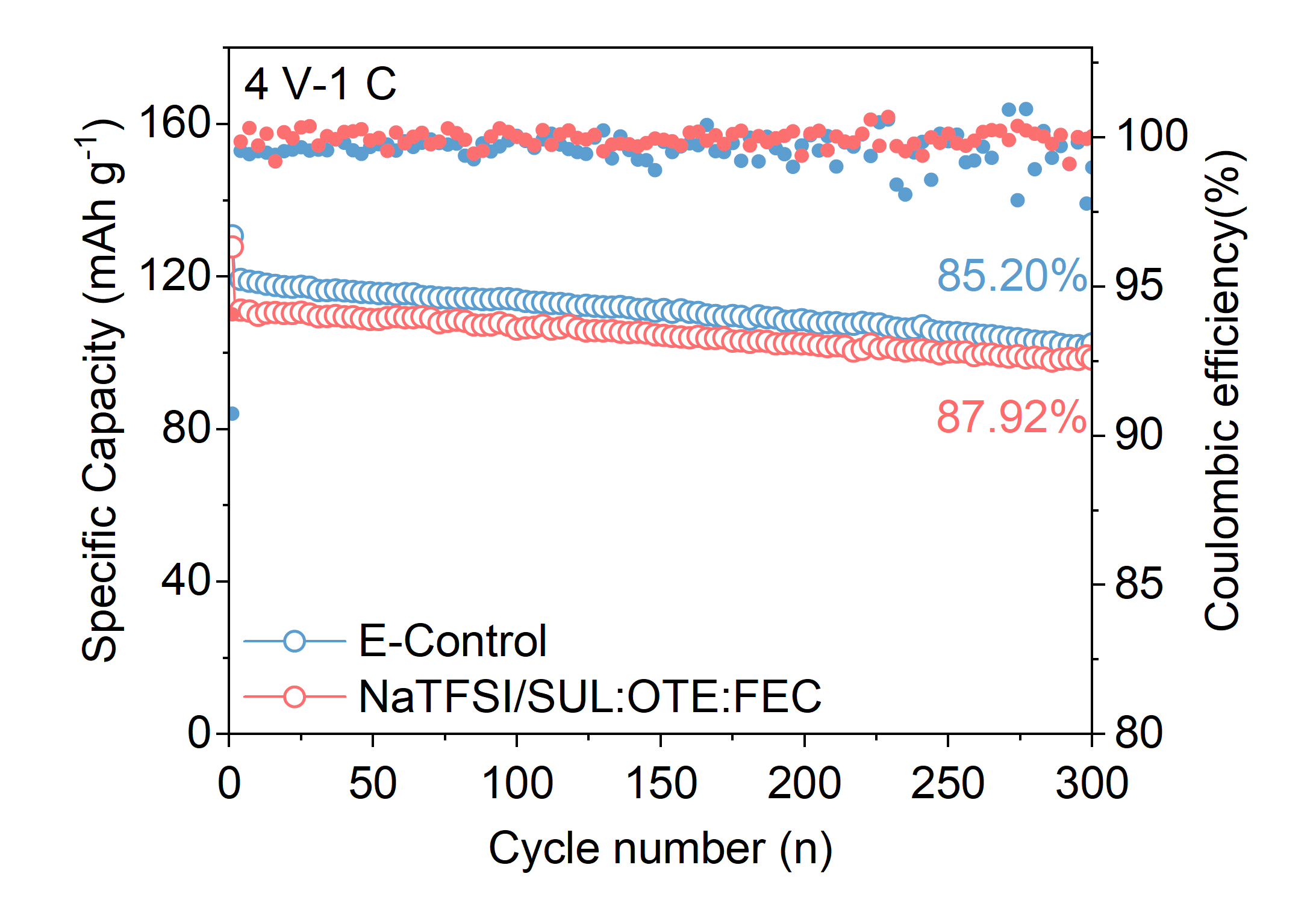


**Fig. S7** Cycling performance of Na||NaNMF cells using E-Control and NaTFSI/SUL:OTE:FEC in the voltage range of 2–4 V at 1 C


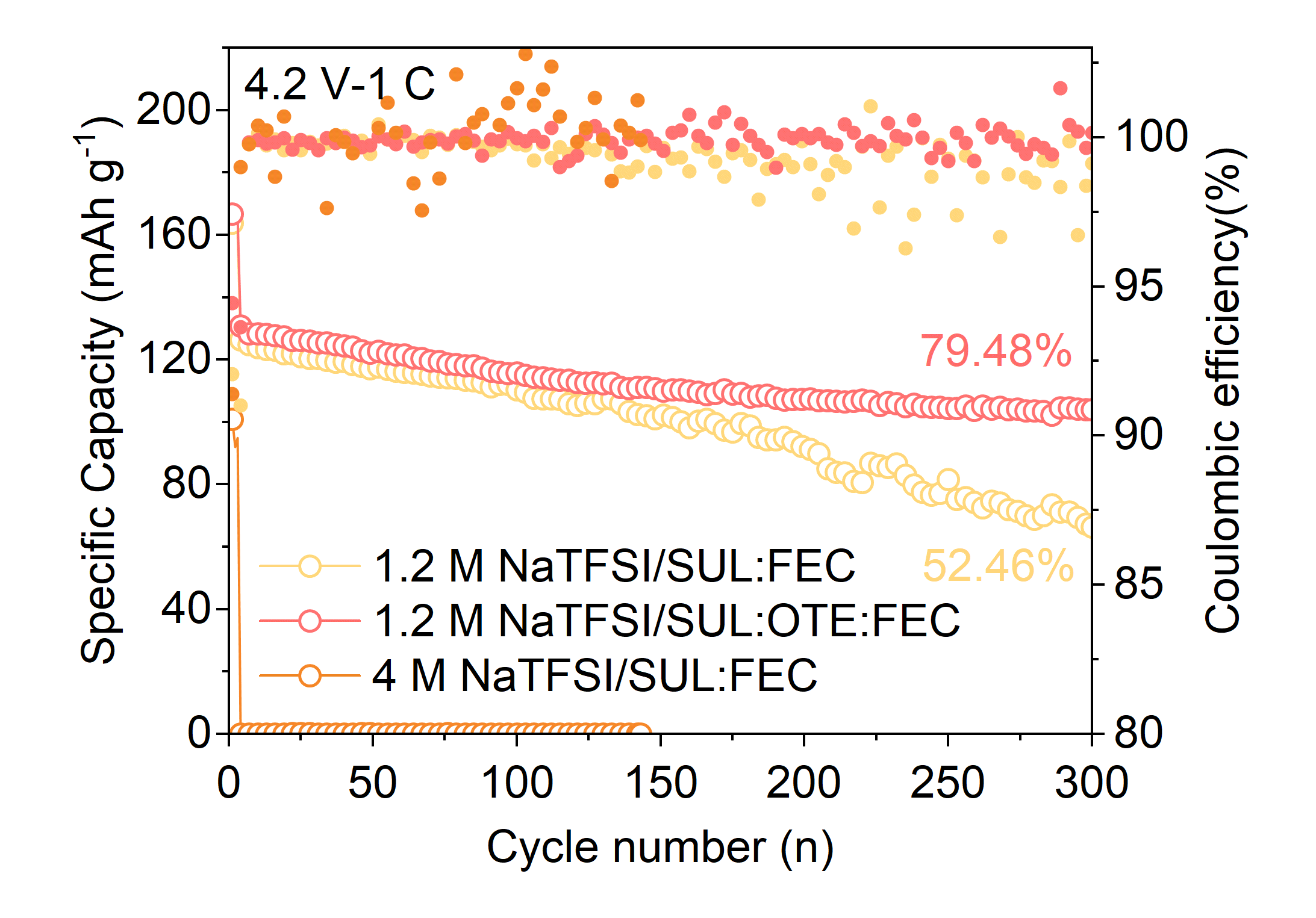


**Fig. S8** Cycling performance of Na||NaNMF cells using 1.2 M NaTFSI/SUL:OTE:FEC, 1.2 M NaTFSI/SUL:FEC and 4 M NaTFSI/SUL:FEC in the voltage range of 2–4.2 V at 1 C


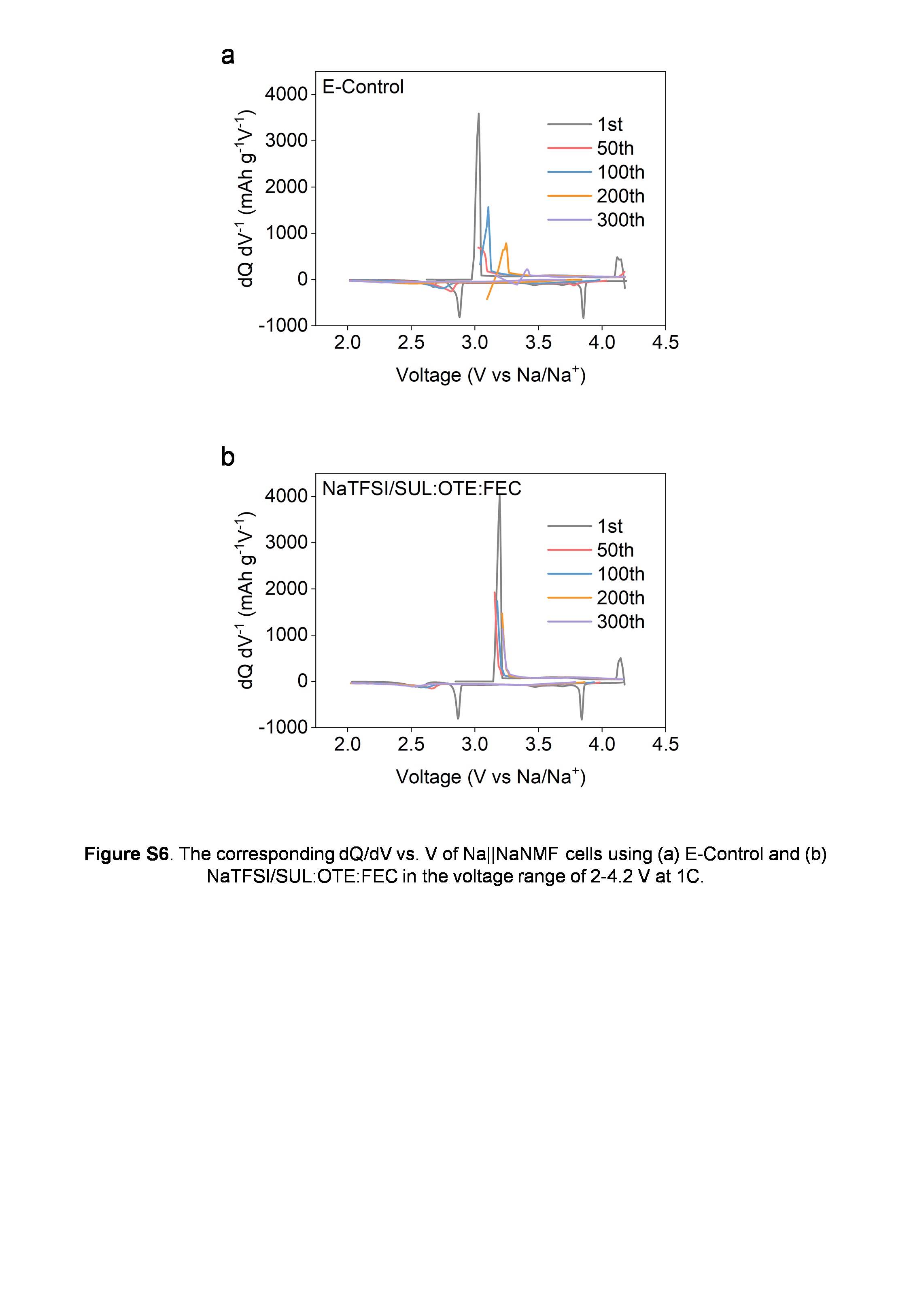


**Fig. S9** The corresponding dQ/dV vs. V of Na||NaNMF cells using (**a**) E-Control and (**b**) NaTFSI/SUL:OTE:FEC in the voltage range of 2–4.2 V


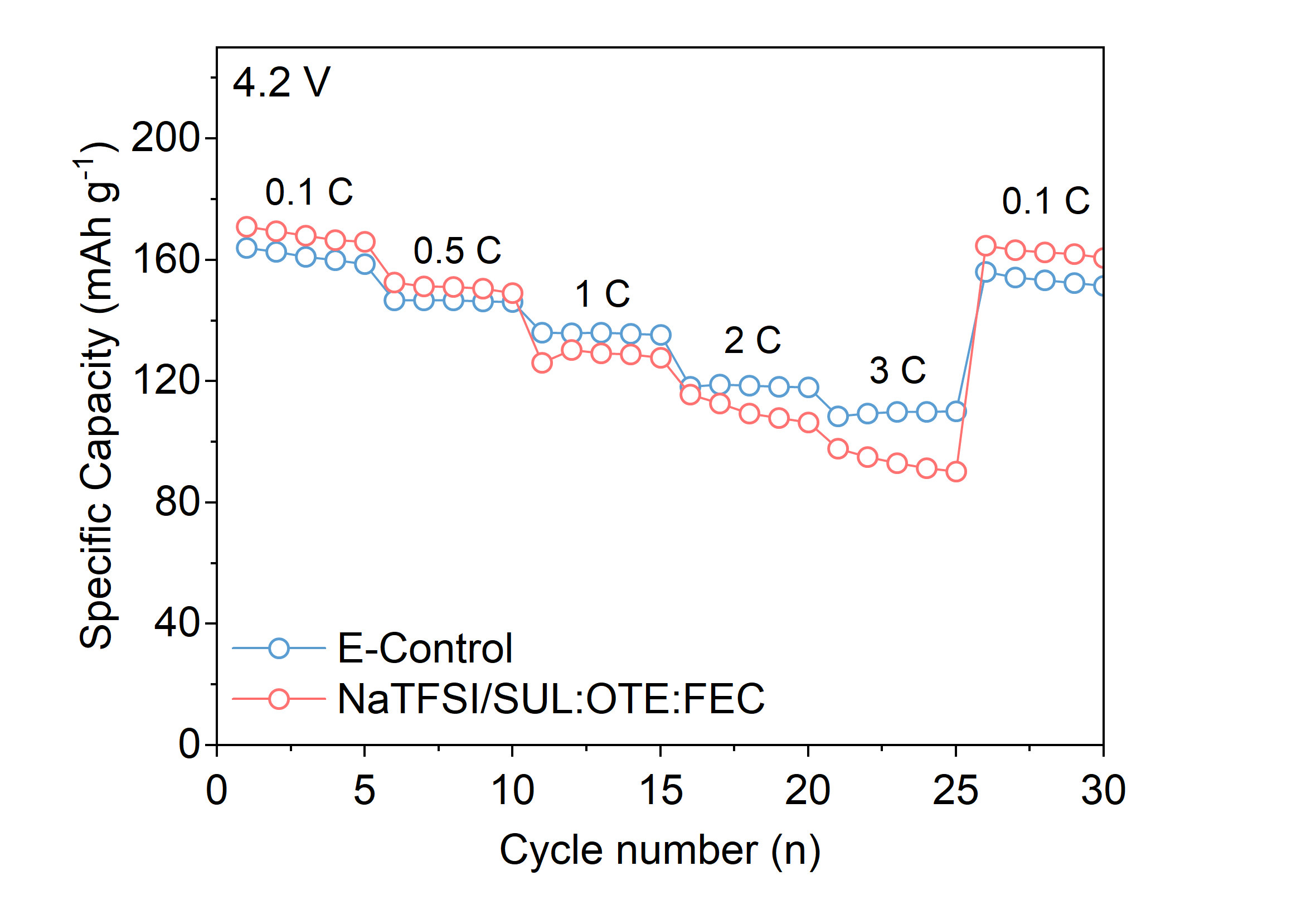


**Fig. S10** Rate capability of Na||NaNMF cells using E-Control and NaTFSI/SUL:OTE:FEC in the voltage range of 2–4.2 V


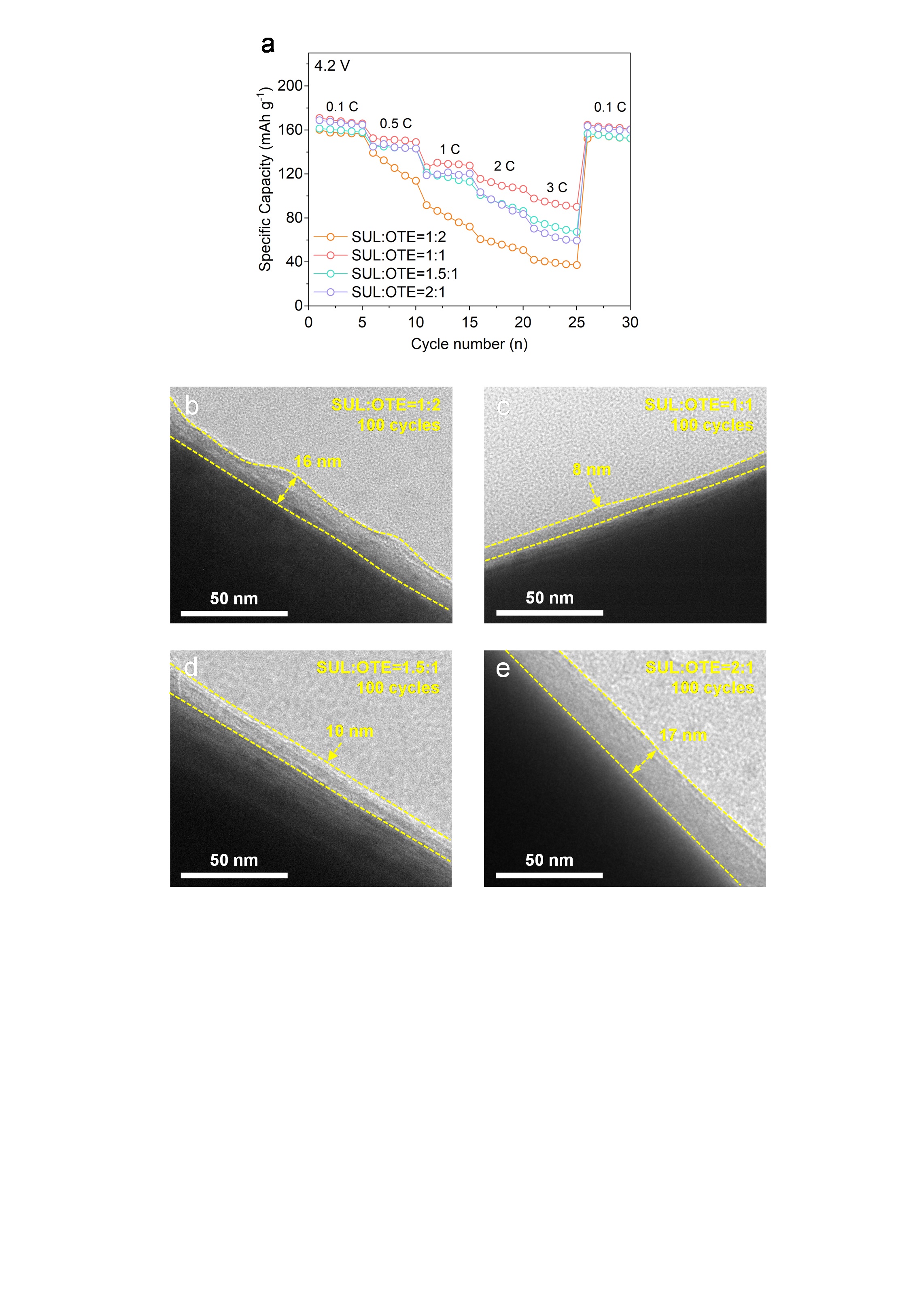


**Fig. S11** (**a)** Rate capability of Na||NaNMF cells using electrolytes with different SUL:OTE molar ratios in the voltage range of 2–4.2 V. (**b–e)** TEM images of NaNMF cathode after 100 cycles in electrolytes with different SUL:OTE molar ratios


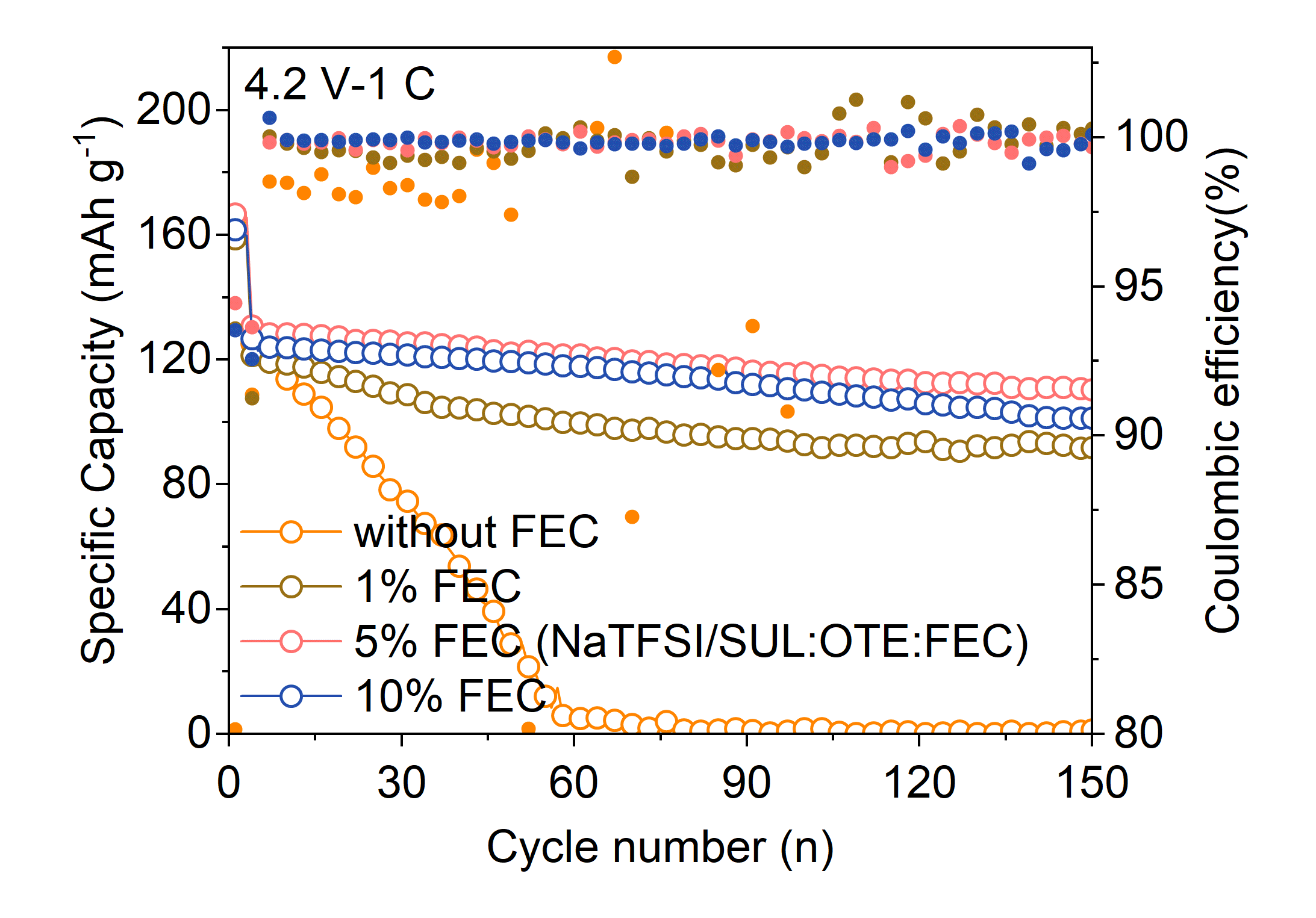


**Fig. S12** Cycling performance of the Na||NaNMF cells using different electrolytes in the voltage range of 2–4.2 V at 1 C


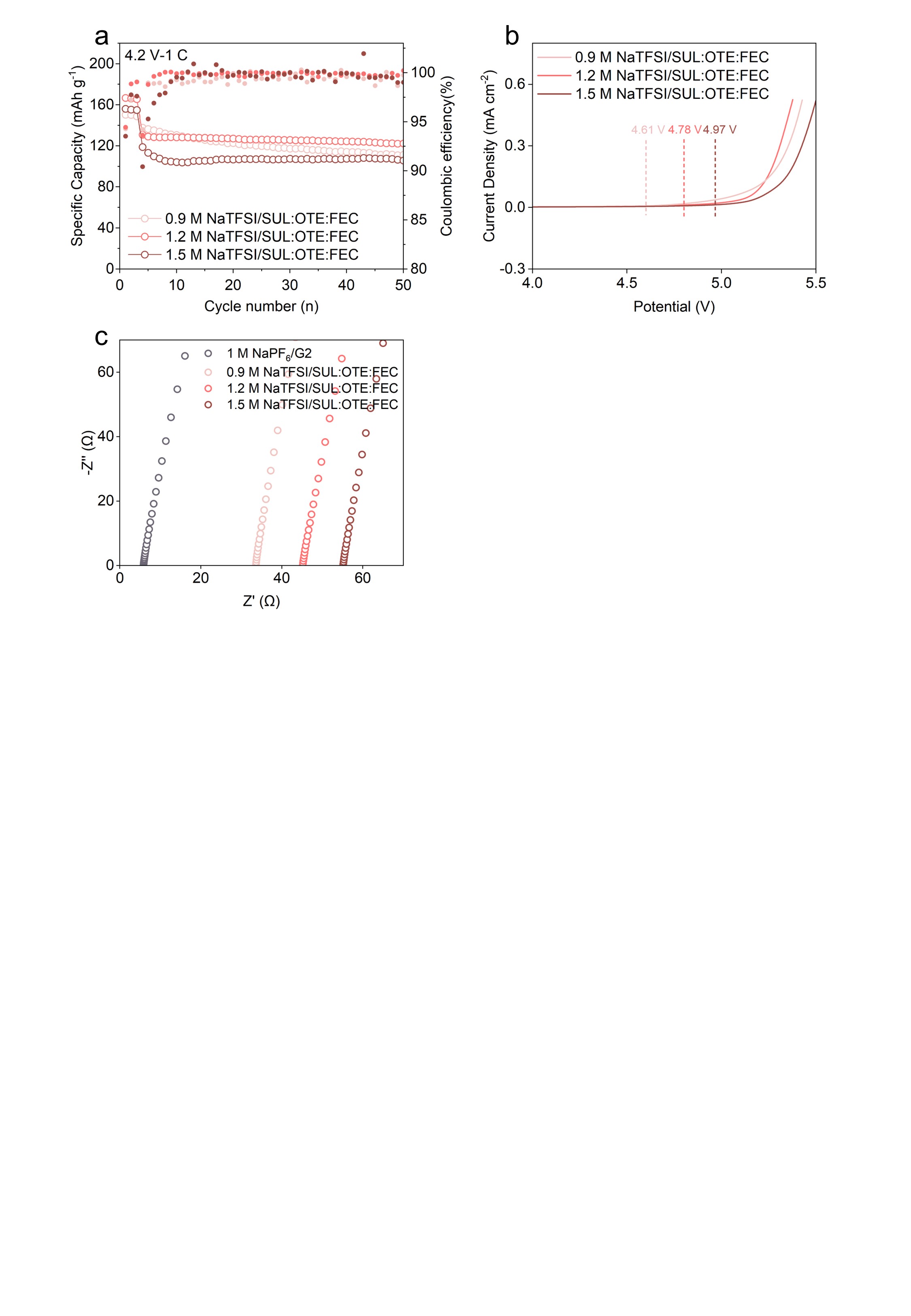


**Fig. S13** (**a)** Cycling performance of the Na||NaNMF cells using different electrolytes in the voltage range of 2–4.2 V at 1 C. **(b)** LSV curves of the Na||steel half-cells with different electrolytes. **(c)** Nyquist plots of symmetric stainless steel cells assembled with different electrolytes


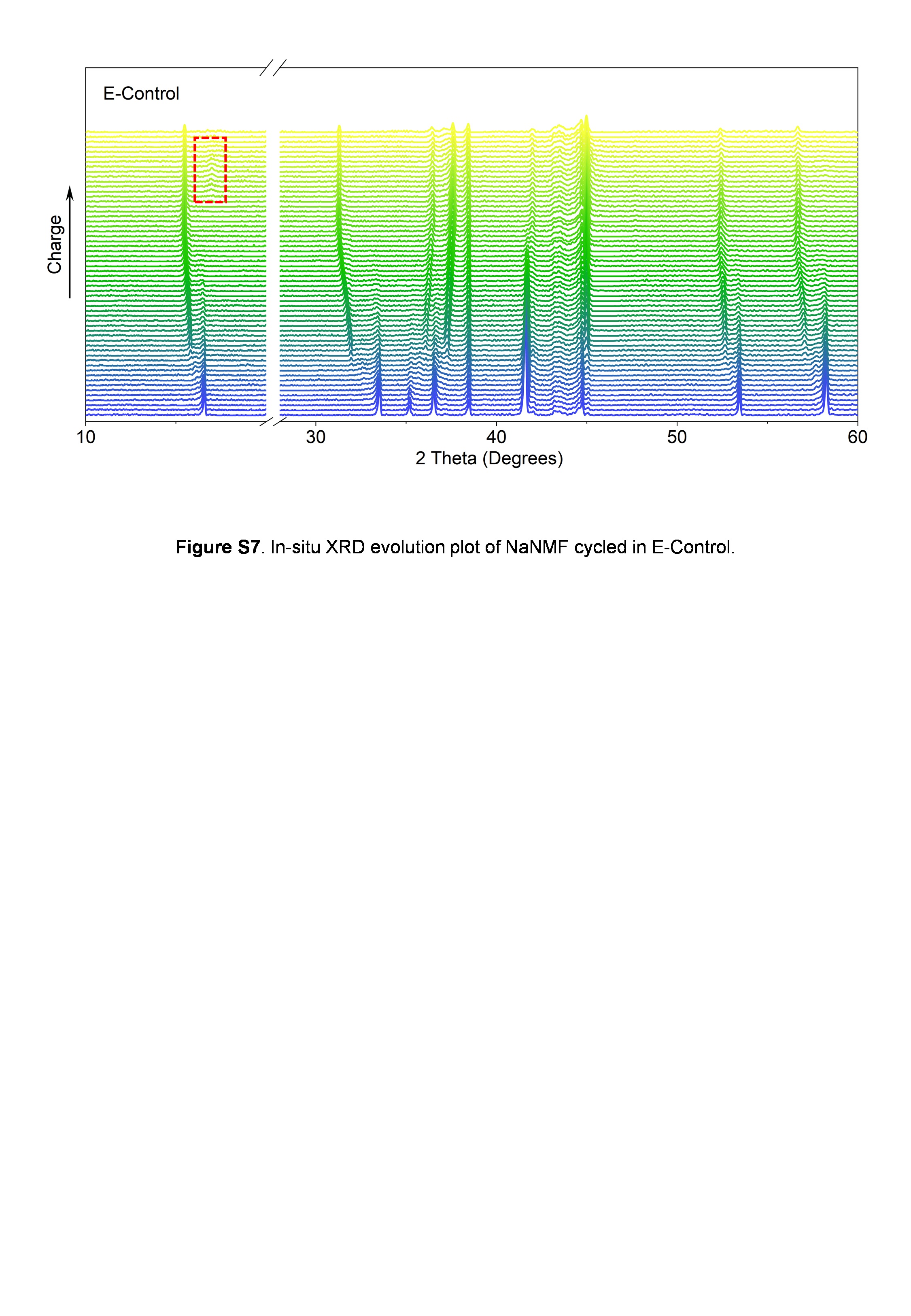


**Fig. S14** In-situ XRD evolution plot of NaNMF cycled in E-Control


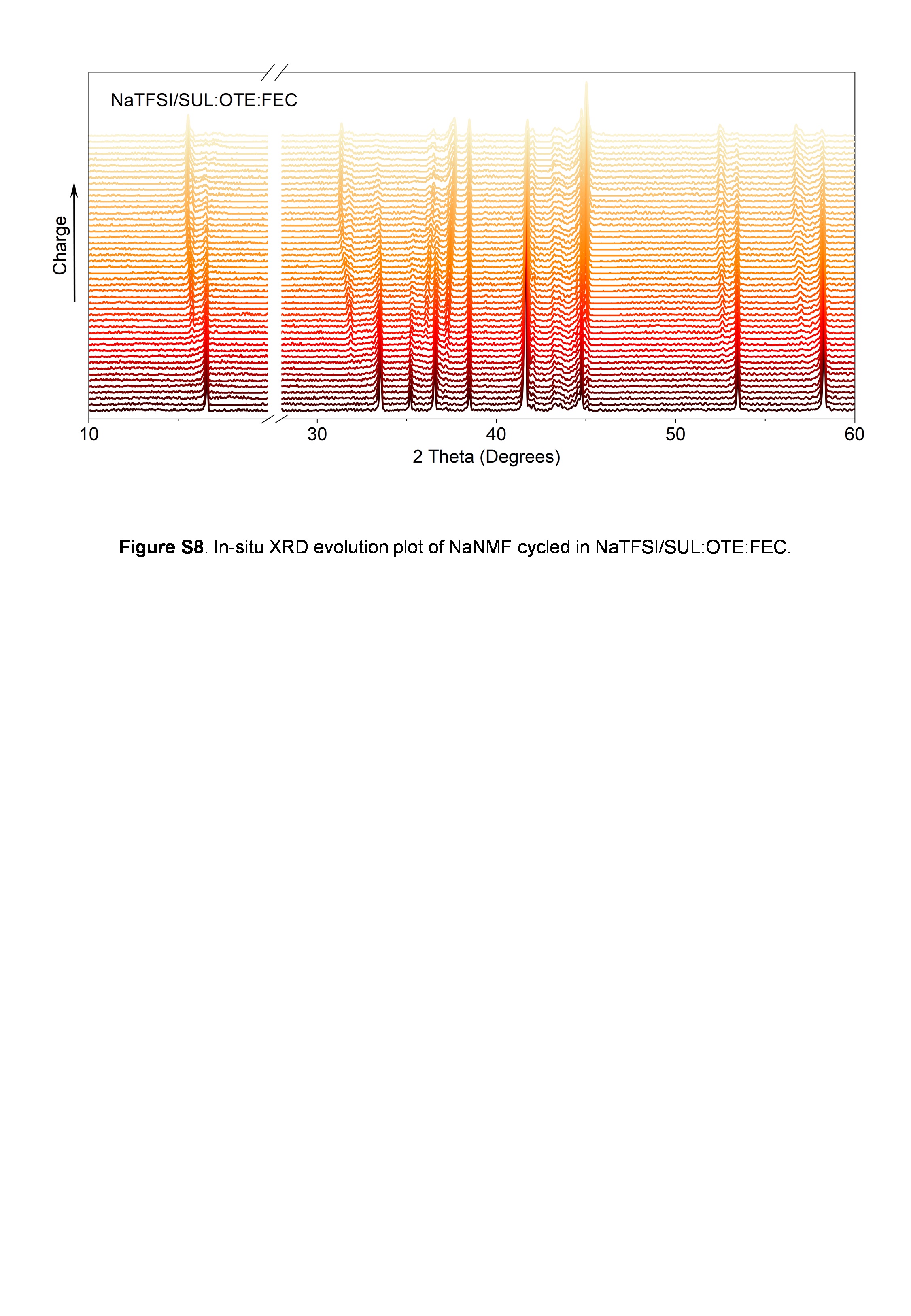


**Fig. S15** In-situ XRD evolution plot of NaNMF cycled in NaTFSI/SUL:OTE:FEC


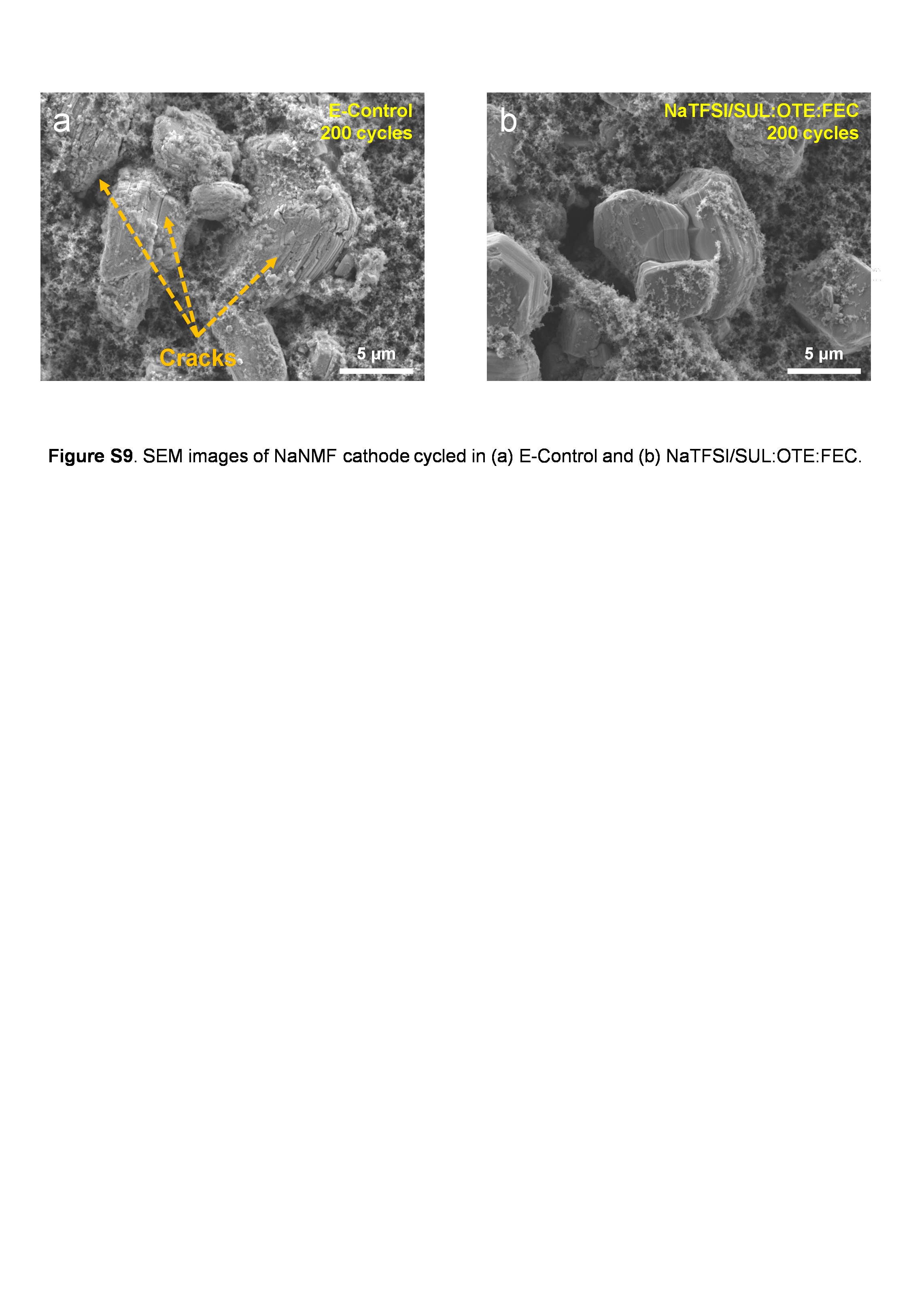


**Fig. S16** SEM images of NaNMF cathode cycled in (**a**) E-Control and (**b**) NaTFSI/SUL:OTE:FEC


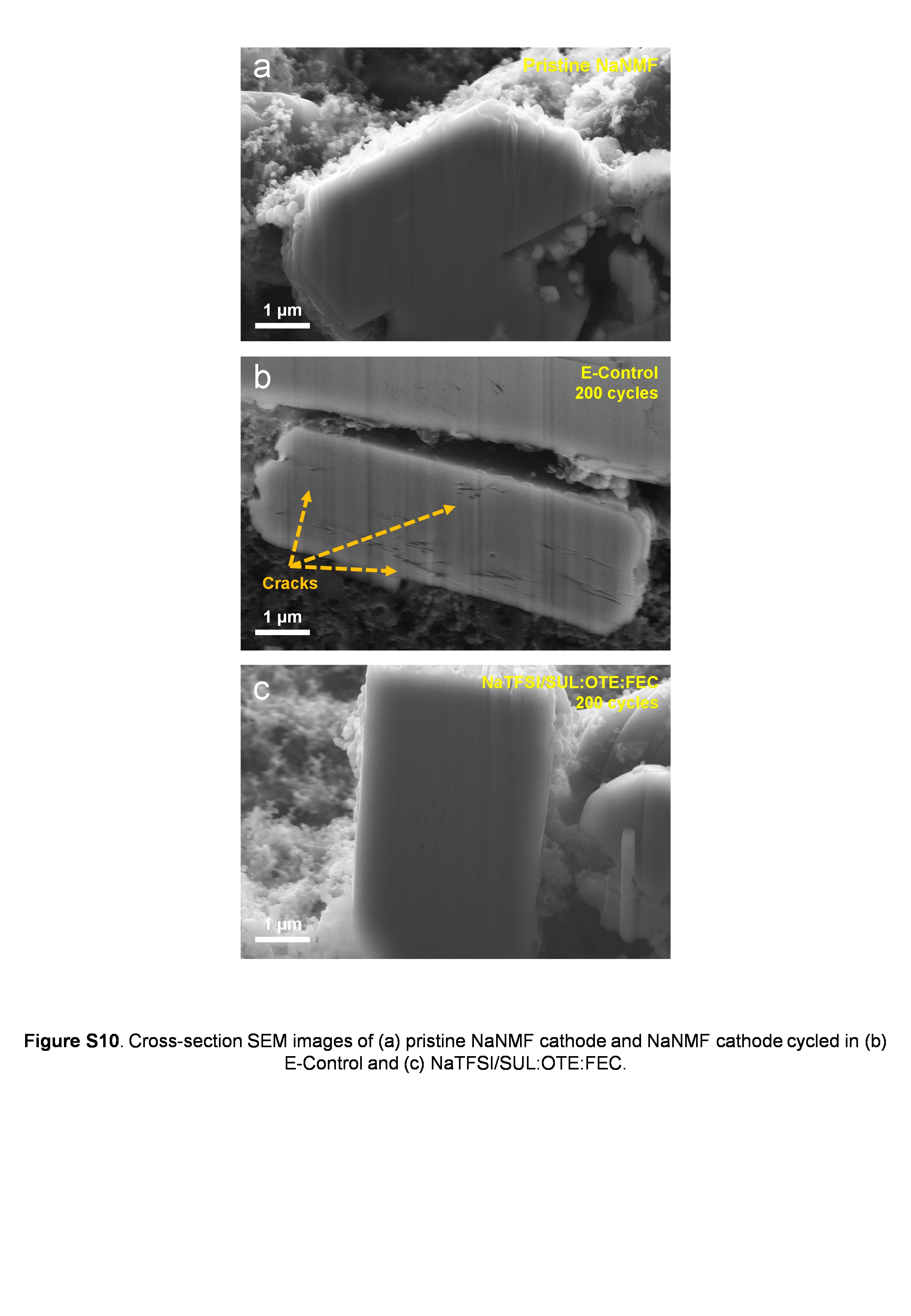


**Fig. S17** Cross-section SEM images of (**a**) pristine NaNMF cathode and NaNMF cathode cycled in (**b**) E-Control and (**c**) NaTFSI/SUL:OTE:FEC


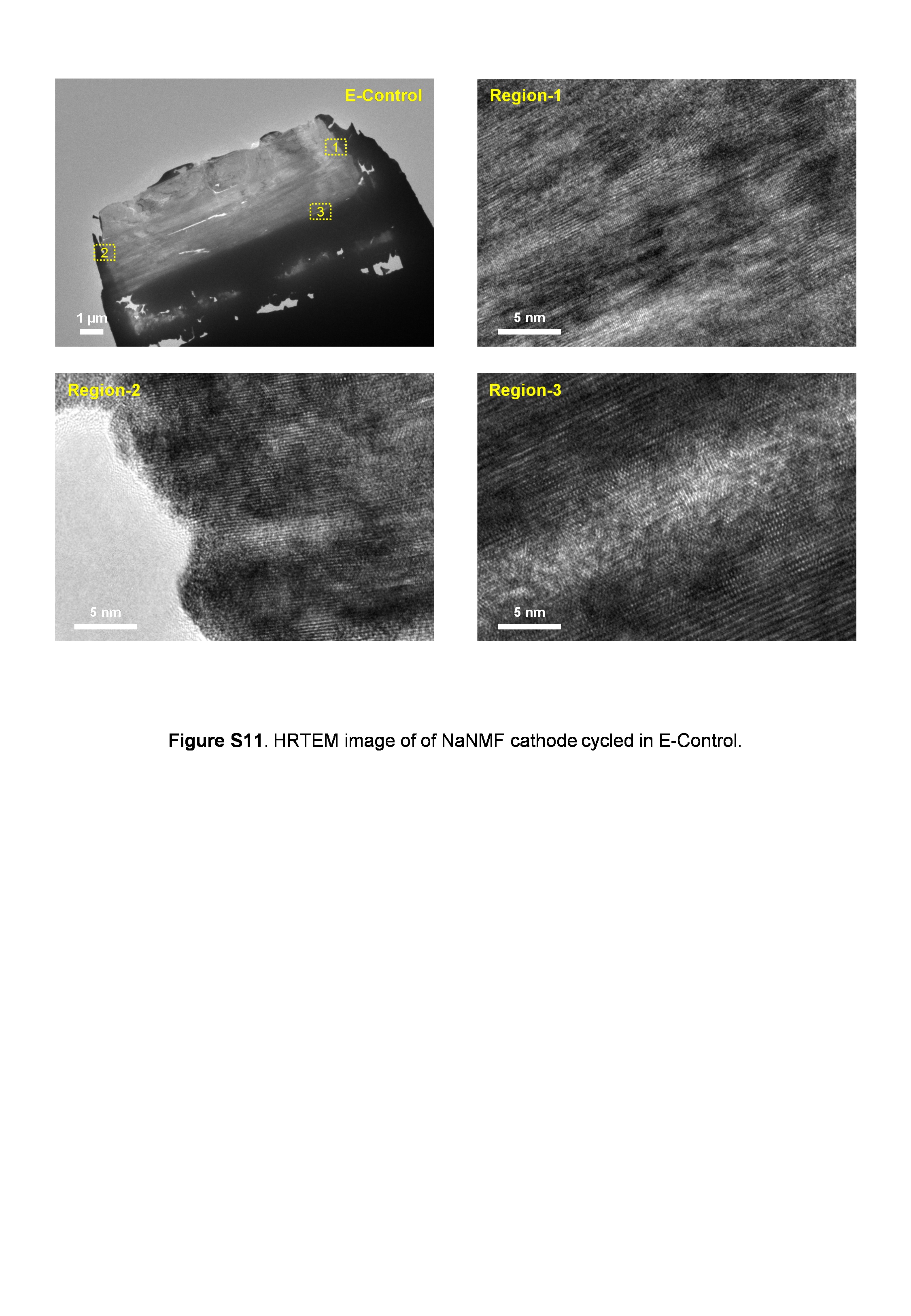


**Fig. S18** HRTEM images of NaNMF cathode cycled in E-Control


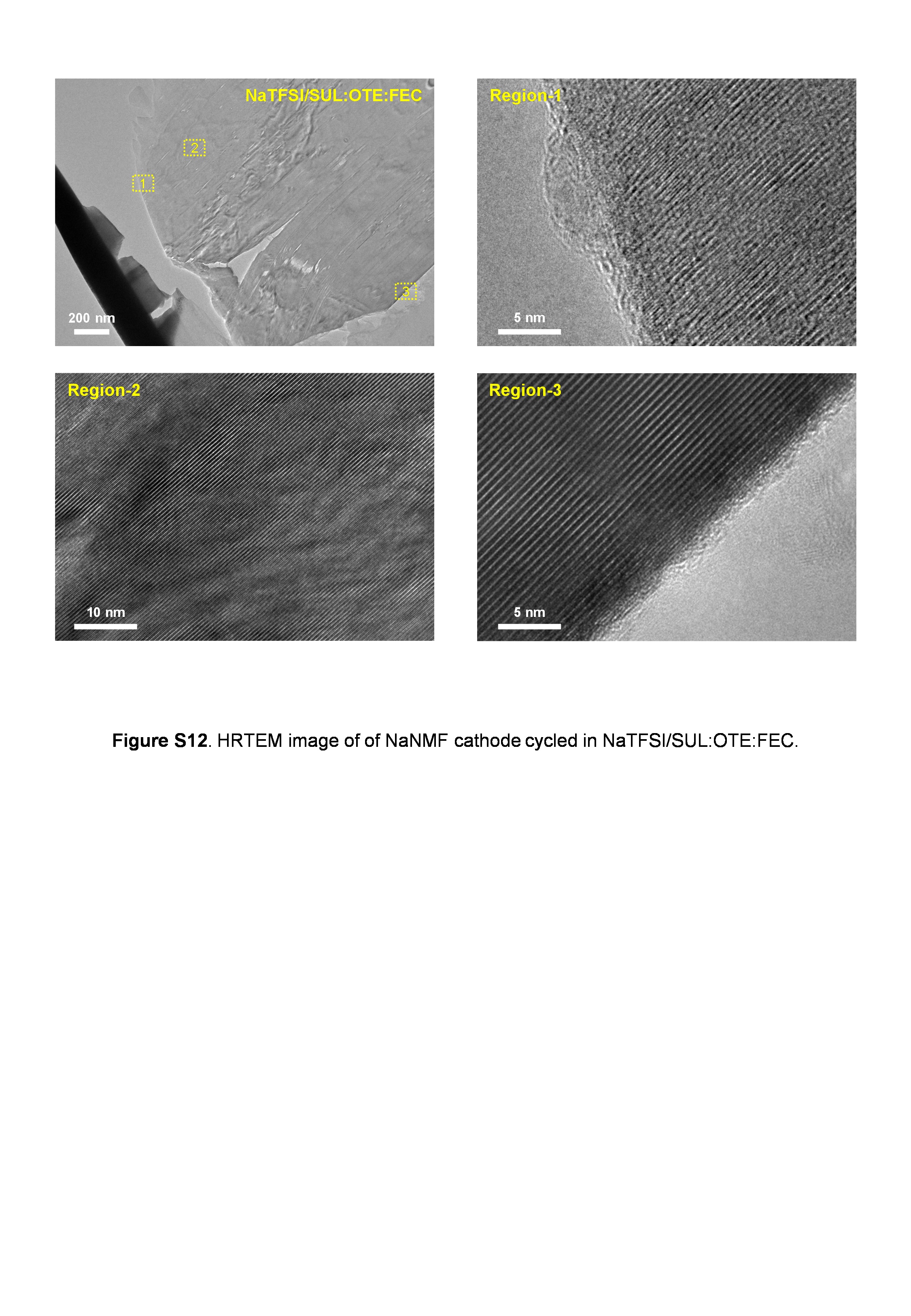


**Fig. S19** HRTEM images of NaNMF cathode cycled in NaTFSI/SUL:OTE:FEC


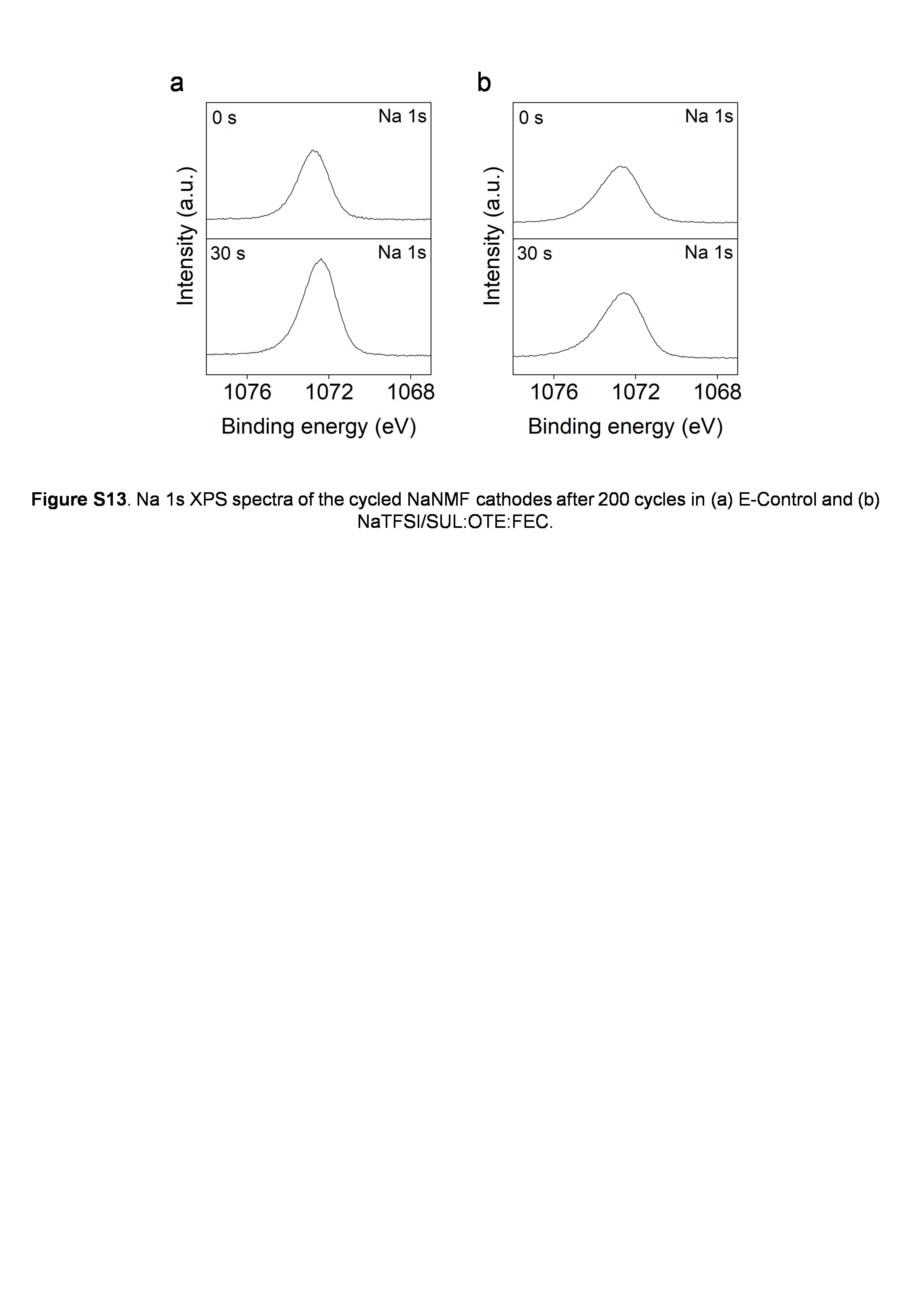


**Fig. S20** Na 1s XPS spectra of the cycled NaNMF cathodes after 200 cycles in (**a**) E-Control and (**b**) NaTFSI/SUL:OTE:FEC


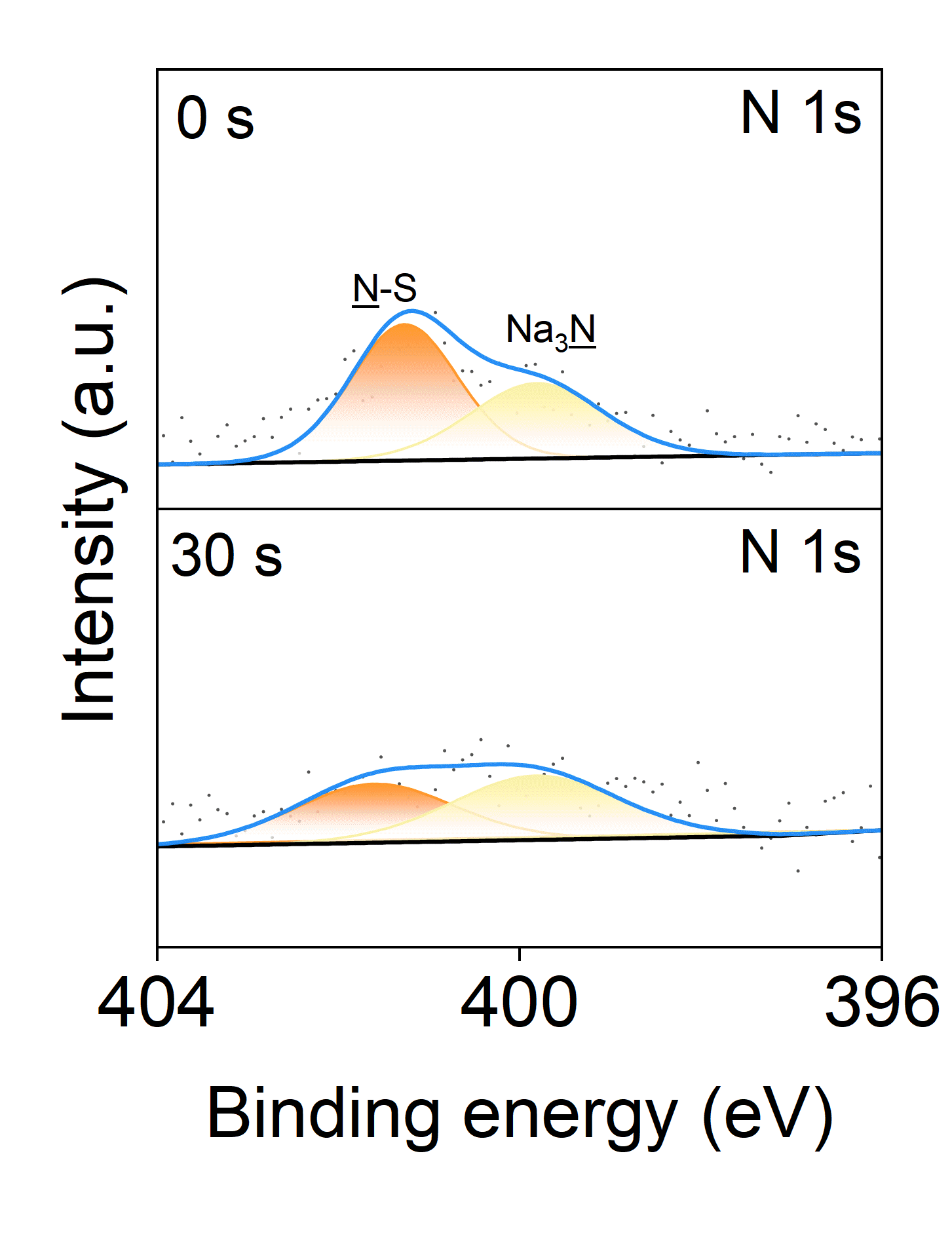


**Fig. S21** N 1s XPS spectra of the cycled NaNMF cathodes after 200 cycles in NaTFSI/SUL:OTE:FEC


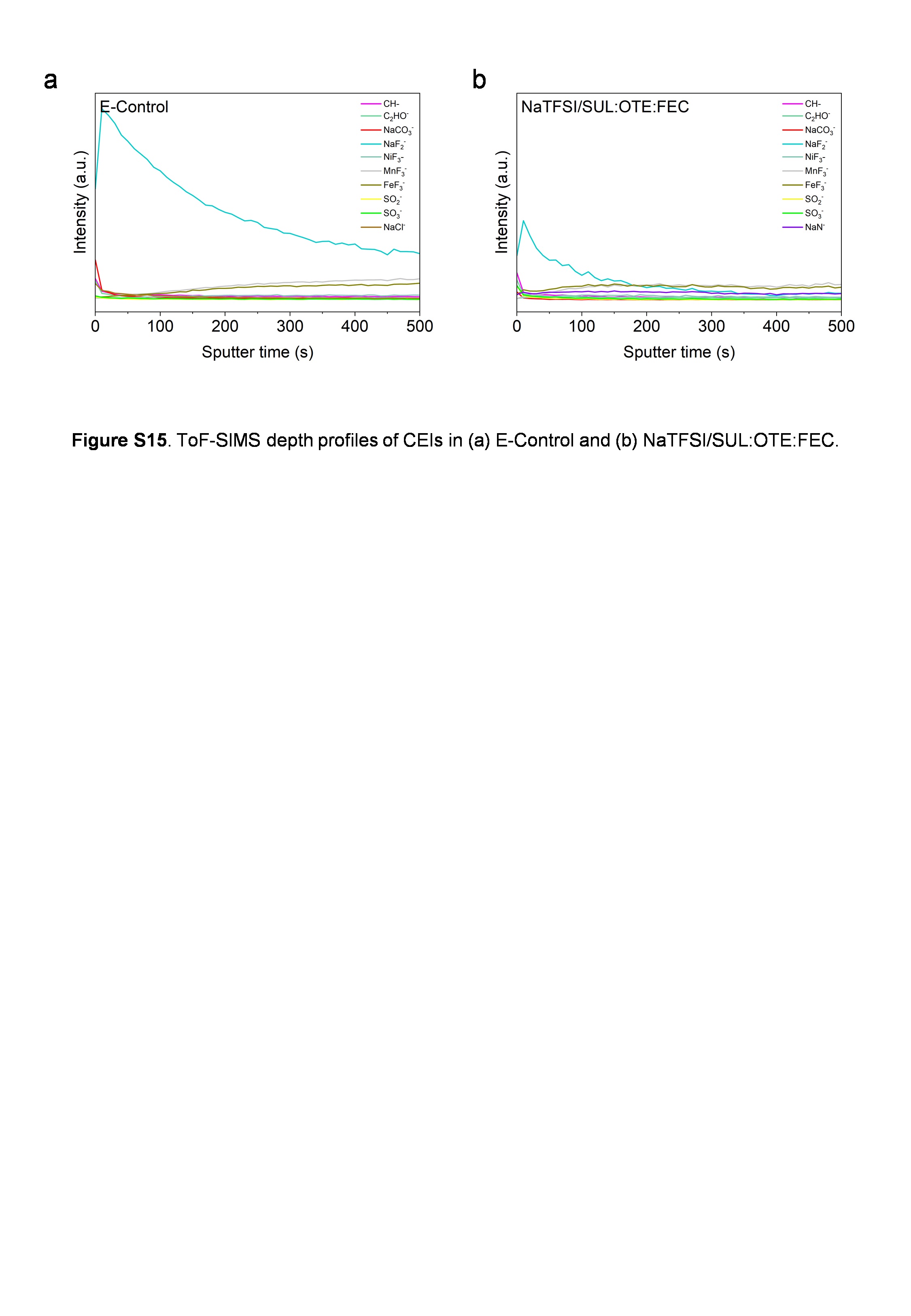


**Fig. S22** TOF-SIMS depth profiles of CEIs in (**a**) E-Control and (**b**) NaTFSI/SUL:OTE:FEC


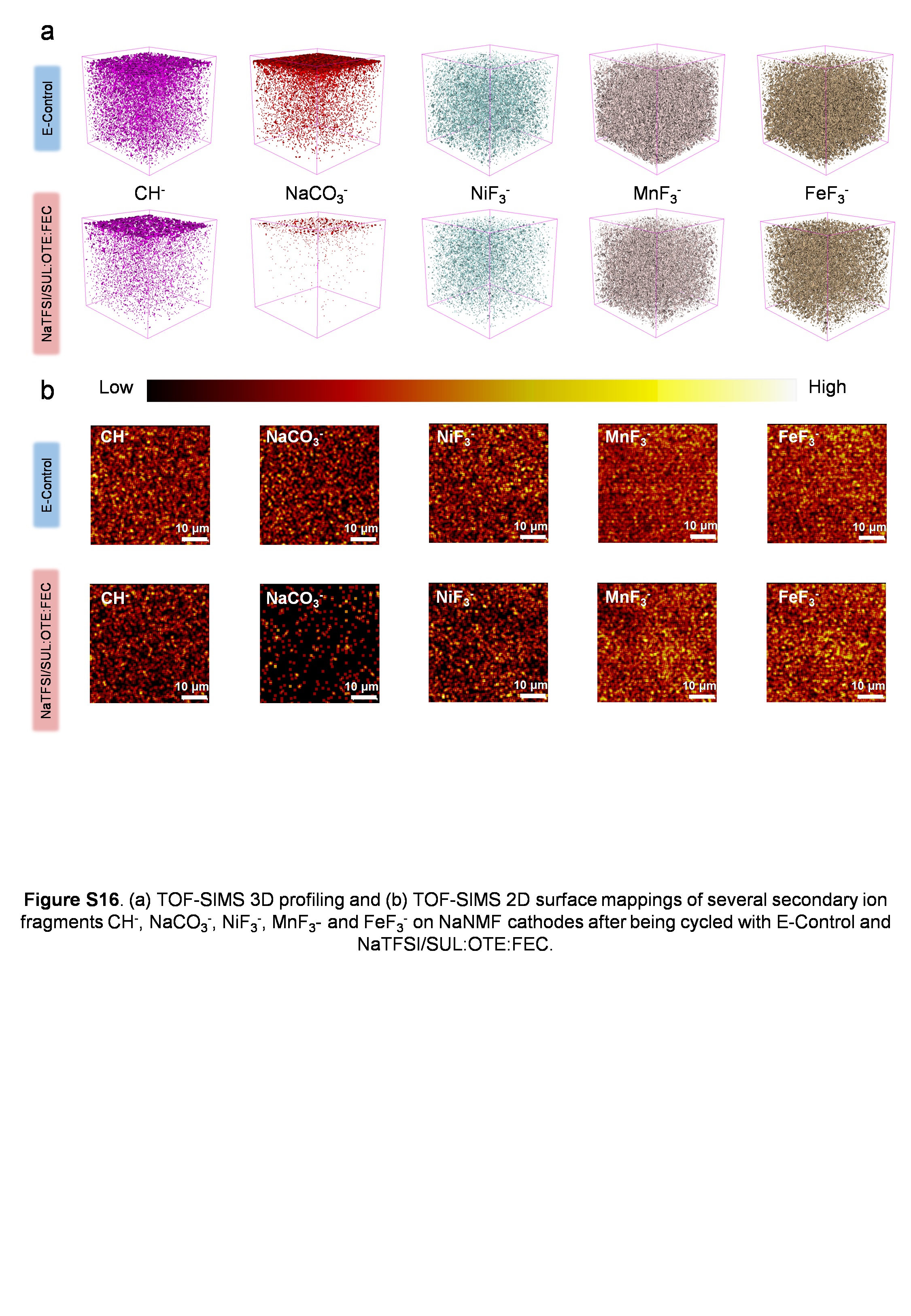


**Fig. S23** (**a**) TOF-SIMS 3D profiling and (**b**) TOF-SIMS 2D surface mappings of several secondary ion fragments SO_2_^-^, SO_3_^-^, NaCl^-^, NaN^-^, MnF_3_^-^ and FeF_3_^-^ on NaNMF cathodes after being cycled with E-Control and NaTFSI/SUL:OTE:FEC


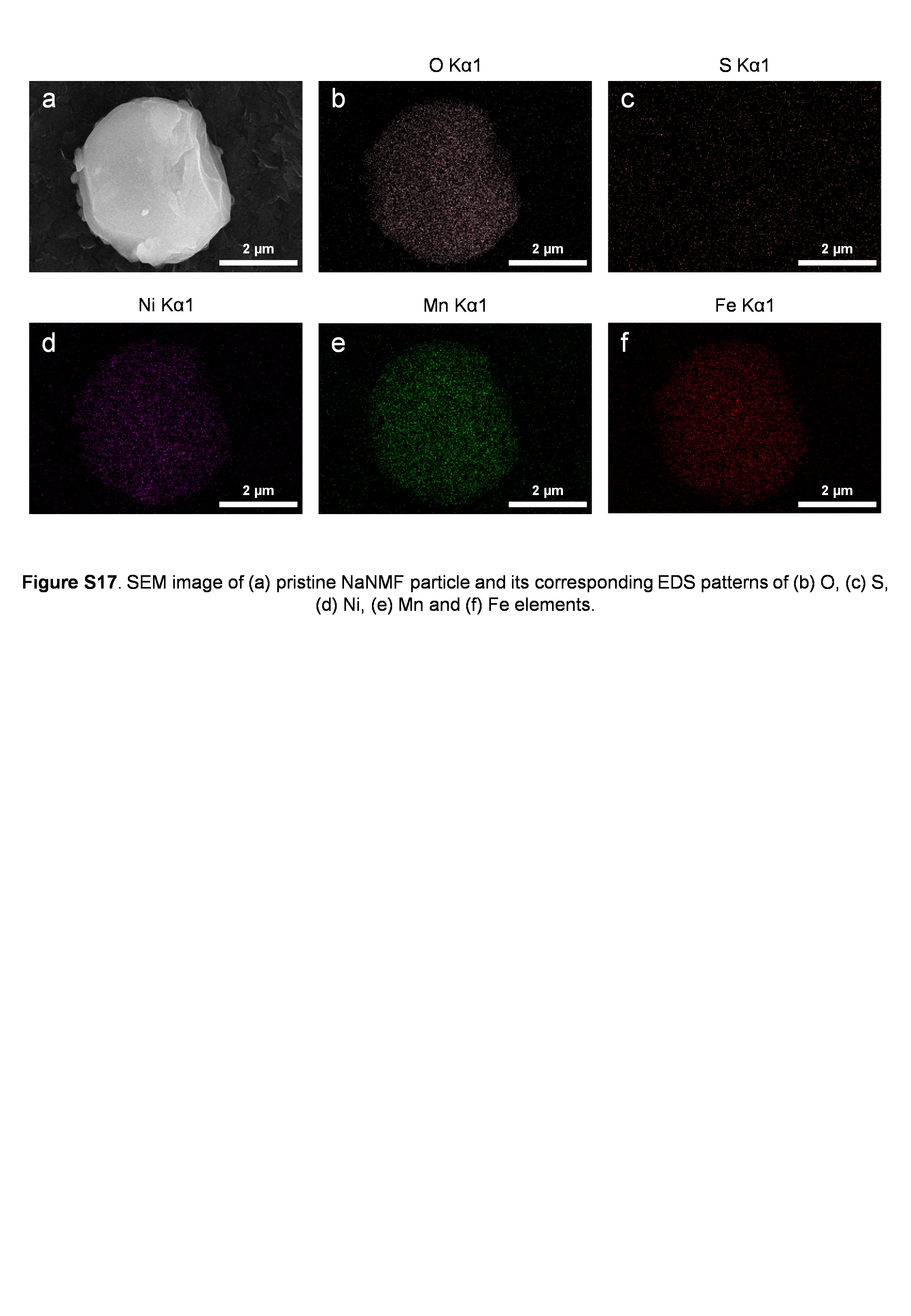


**Fig. S24** SEM images of (**a**) pristine NaNMF particle and its corresponding EDS patterns of (**b**) O, (**c**) S, (**d**) Ni, (**e**) Mn and (**f**) Fe elements


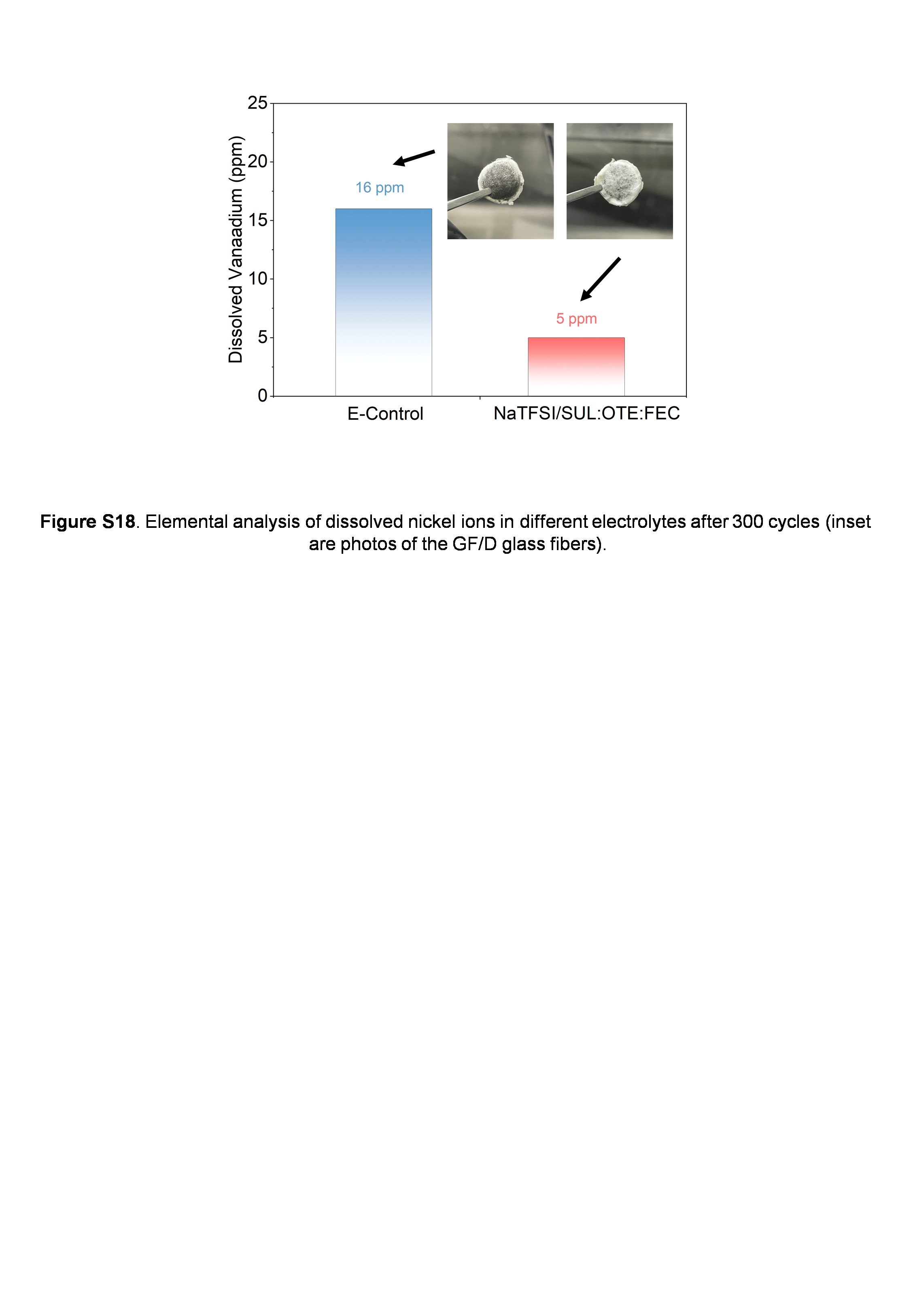


**Fig. S25** Elemental analysis of dissolved nickel ions in different electrolytes after 300 cycles (inset are photos of the GF/D glass fibers)


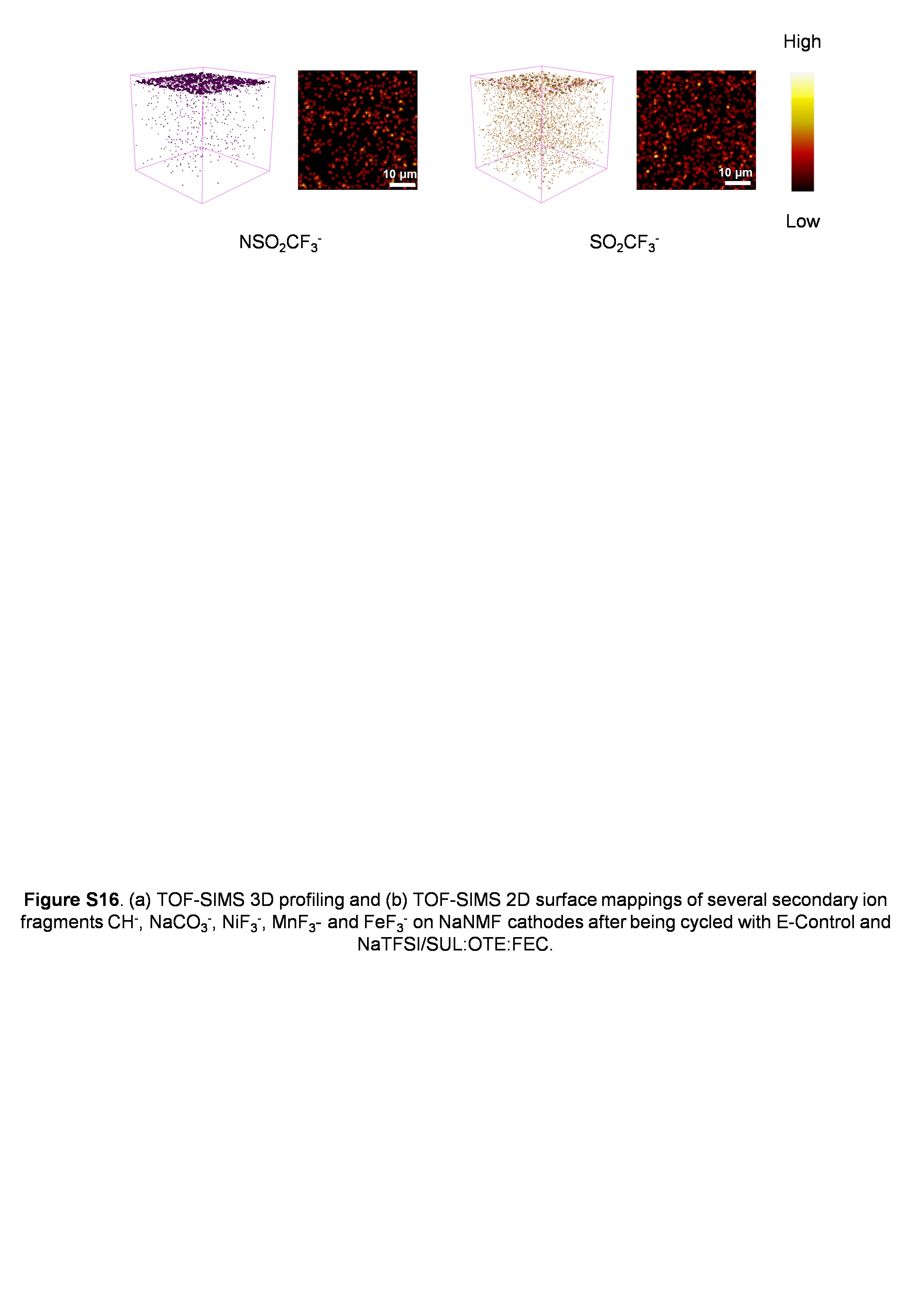


**Fig. S26** TOF-SIMS 3D profiling and TOF-SIMS 2D surface mappings of several secondary ion fragments NSO_2_CF_3_^-^ and SO_2_CF_3_^-^ on NaNMF cathodes after being cycled with NaTFSI/SUL:OTE:FEC


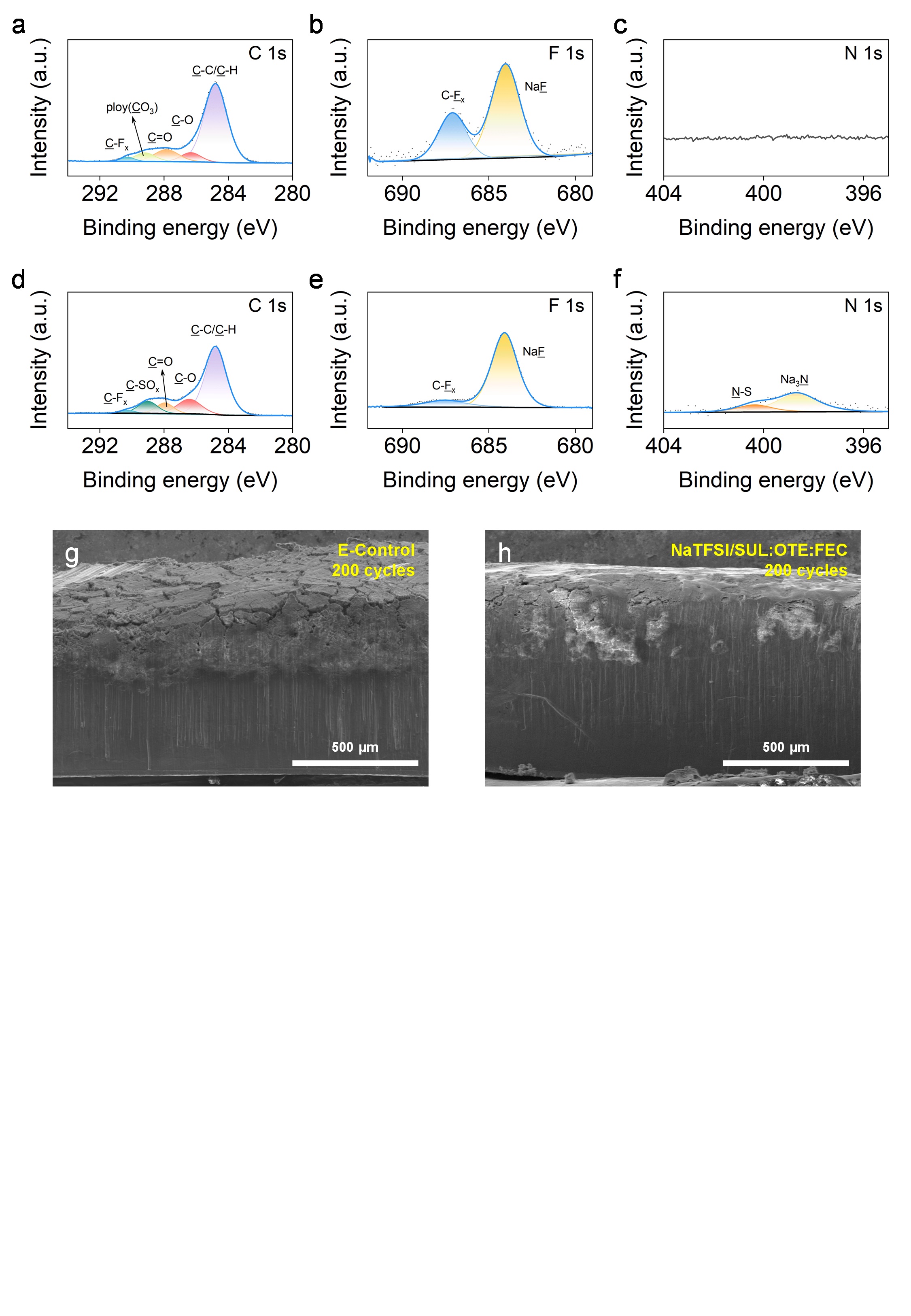


**Fig. S27** XPS spectra of the cycled Cu Foil after 100 cycles in (**a–c**) E-Control. XPS spectra of the cycled Cu foil after 100 cycles in (**d–f**) NaTFSI/SUL:OTE:FEC. Cross-section SEM images of Na anode cycled in (**g**) E-Control and (**h**) NaTFSI/SUL:OTE:FEC


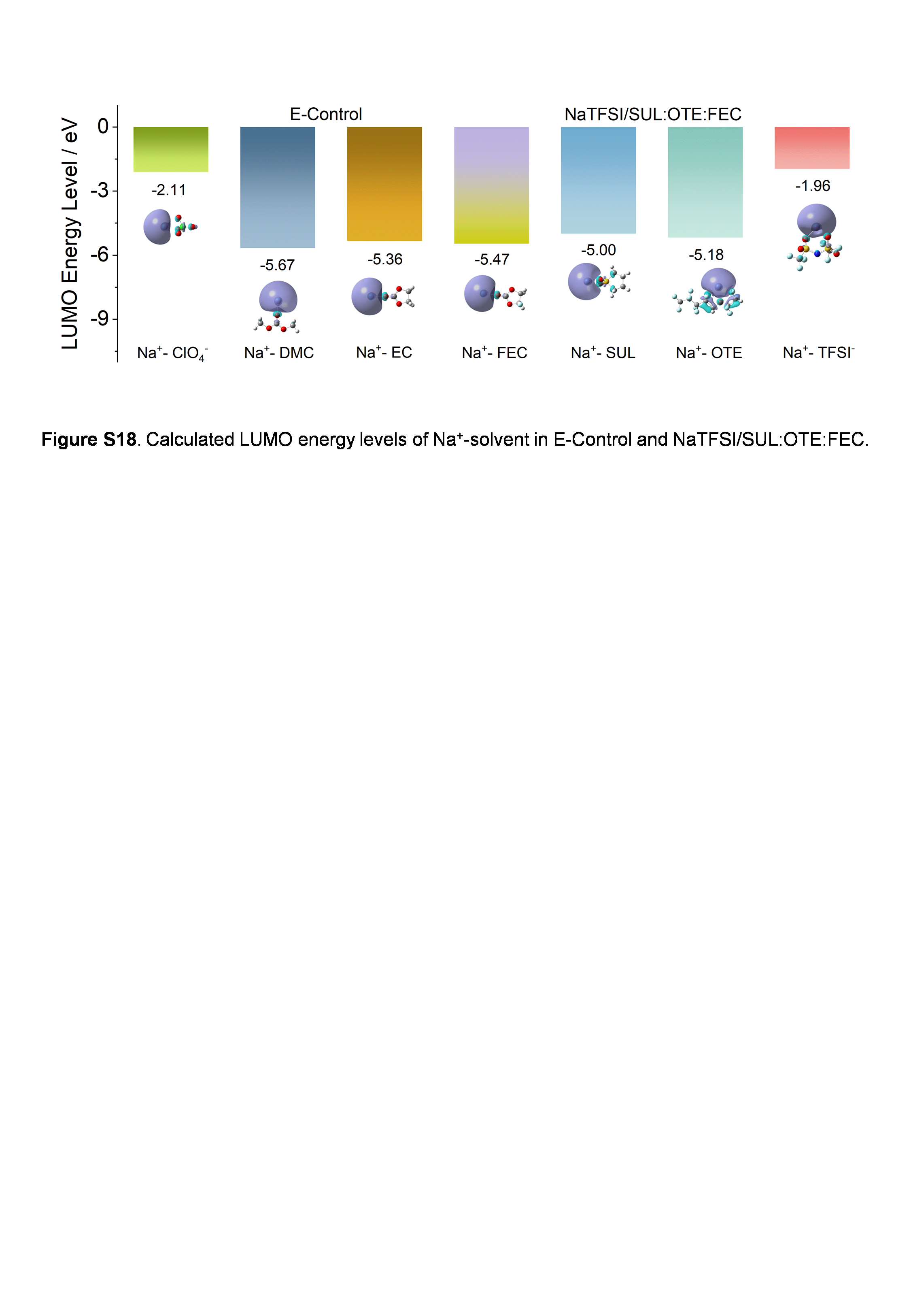


**Fig. S28** Calculated LUMO energy levels of Na^+^-anion and Na^+^-solvent in E-Control and NaTFSI/SUL:OTE:FEC


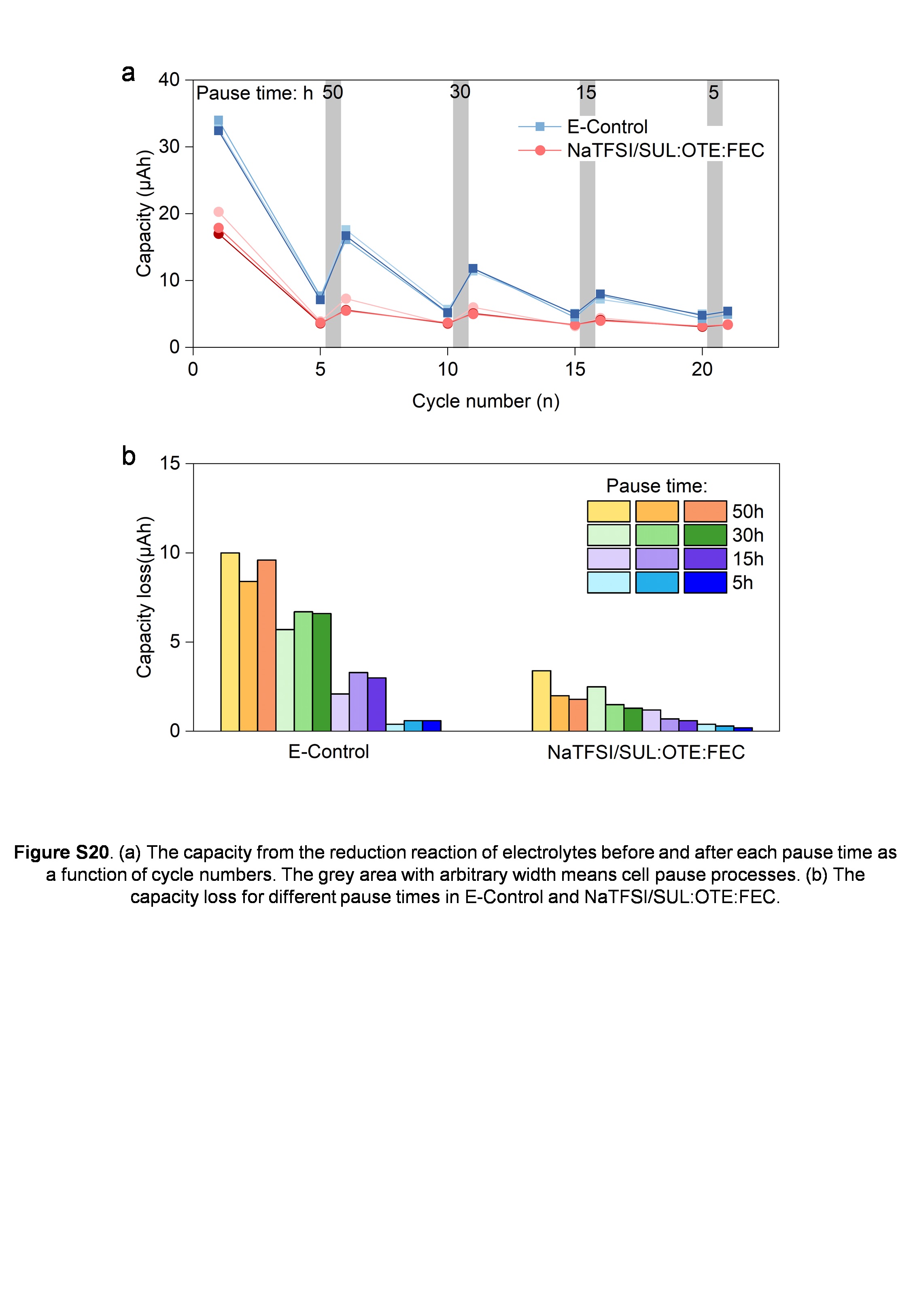


**Fig. S29** (**a**) The capacity from the reduction reaction of electrolytes before and after each pause time as a function of cycle numbers. The grey area with arbitrary width means cell pause processes. (**b**) The capacity loss for different pause times in E-Control and NaTFSI/SUL:OTE:FEC


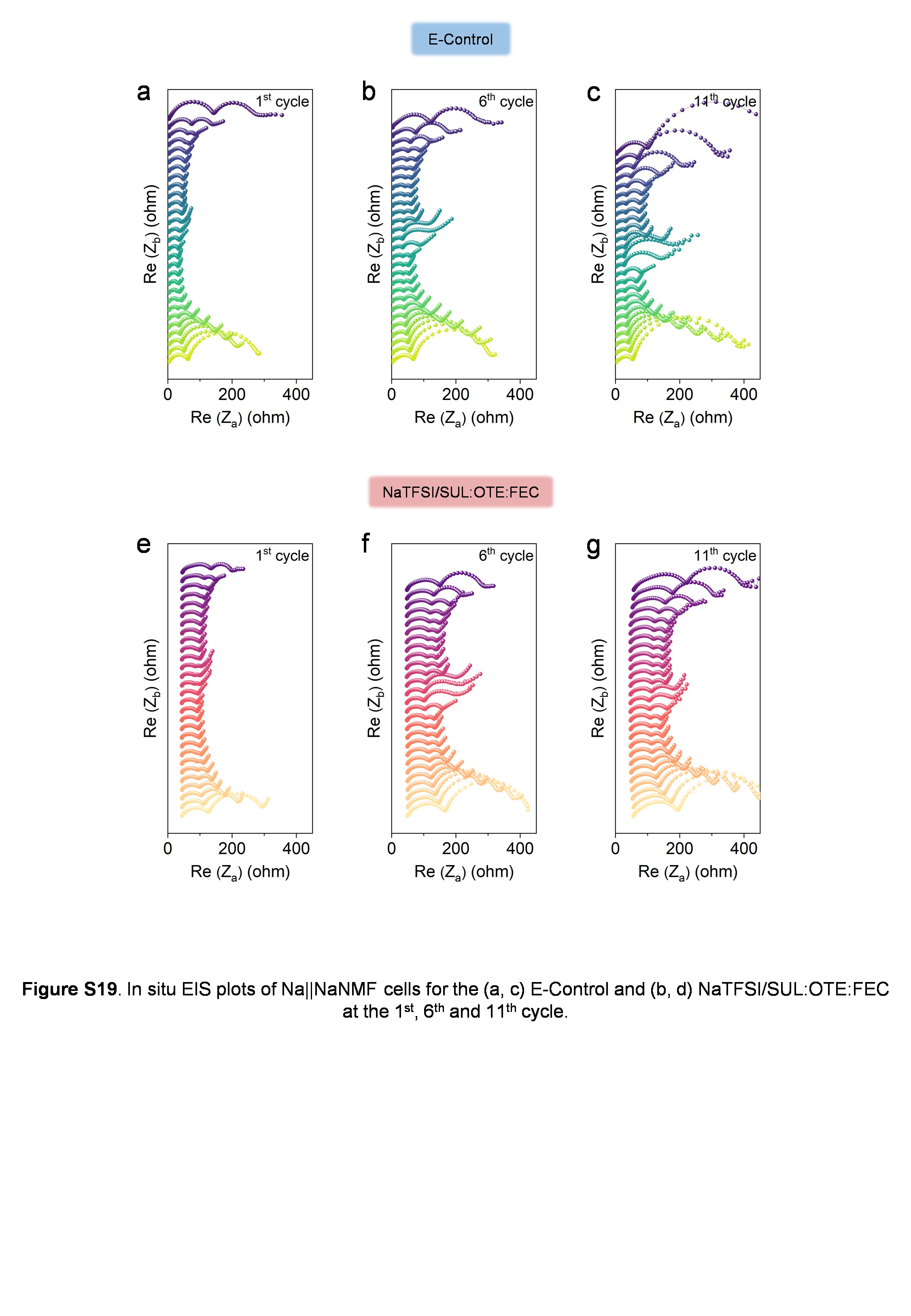


**Fig. S30** In situ EIS plots of Na||NaNMF cells for the (**a-c**) E-Control and (**d-e**) NaTFSI/SUL:OTE:FEC at the 1^st^, 6^th^ and 11^th^ cycle


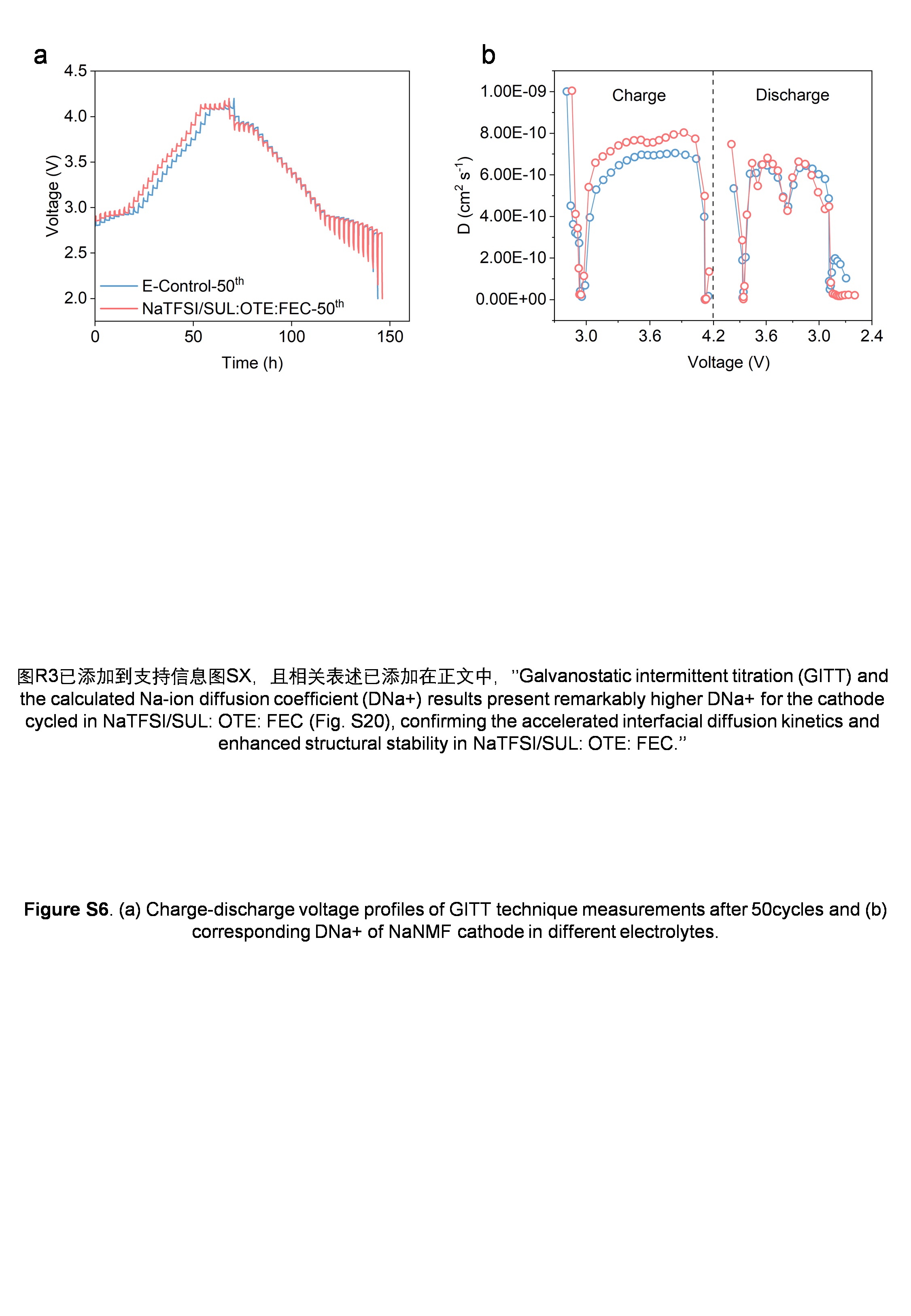


**Fig. S31** (**a**) Charge-discharge voltage profiles of GITT technique measurements after 50 cycles and (**b**) corresponding D_Na+_ of NaNMF cathode in E-Control and NaTFSI/SUL:OTE:FEC

**Table S1** Compositions and physicochemical properties of the electrolytes investigated in this work

| Electrolyte | FEC Addition | M (mol/L) | Conductivity (mS cm^-1^) (25 °C) |
| --- | --- | --- | --- |
| NaTFSI/SUL:OTE:FEC (SUL:OTE 1:1 molar ratio) | 5 wt.% | 1.2 | 0.97 |
| 0.9 M NaTFSI/ SUL:OTE:FEC | 5 wt.% | 0.9 | 1.30 |
| 1.5 M NaTFSI/ SUL:OTE:FEC | 5 wt.% | 1.5 | 0.76 |
| 1.2 M NaTFSI/SUL:FEC | 5 wt.% | 1.2 | 3.08 |
| 4 M NaTFSI/SUL:FEC | 5 wt.% | 4 | 0.20 |
| E-Control (NaClO_4_/DMC:EC:FEC; DMC:EC 1:1 vol. ratio) | 5 wt.% | 1 | 8.42 |

**Table S2** Viscosity of NaTFSI/SUL:OTE:FEC and E-Control

| Electrolyte | Viscosity (mPa S) (28 °C) |
| --- | --- |
| NaTFSI/SUL:OTE:FEC | 20.50 |
| E-Control | 5.72 |

**Table S3** Electrochemical performance comparison between the published literatures and this work

| Electrolyte | System | Cut-off Voltage (V) | Cycle No. | Initial capacity (mAh g^-1^)/ Capacity retention | Flammability | References |
| --- | --- | --- | --- | --- | --- | --- |
| 1 M NaClO_4_ in TEP with 5wt.% FEC | Na\|\|P2- Na_2/3_Cu_2/9_Fe_1/9_Mn_2/3_O_2_ | 1.5–4.6 V | 200, 2 C | About 80/ 76.28% | No | [S1] |
| 1 M NaPF_6_ EMC/FEC/TTE in a 6:1:3 vol ratio | Na\|\|P2- Na_0.7_Li_0.03_Mg_0.03_Ni_0.27_Mn_0.6_Ti_0.07_O_2_ | 2.2–4.4 V | 200, 1 C | 129/ 87.3% | Yes | [S2] |
| 1 M NaClO_4_ in PC with 2 wt.% FEC and 2 wt.% DGA | Na\|\|P2- Na_0.67_Li_0.2_Ni_0.23_Mn_0.67_O_2_ | 2–4.3 V | 500, 0.5 C | 96.82/ 91.56% | Yes | [S3] |
| 1 M NaClO_4_ in PC with 5 vol.% FEC and 3 wt.% SA | Na\|\|P2- Na_0.6_Li_0.15_Ni_0.15_Mn_0.55_Cu_0.15_O_2_ | 2–4.5 V | 400, 1 C | About 100/ 87.2% | Yes | [S4] |
| 1 M NaPF_6_ PC/EMC in a 1:1 vol ratio with 4 vol.% FEC and 0.5 wt.% AIBN | Na\|\|P2/O3- Na_0.67_Li_0.16_Ni_0.33_Mn_0.67_O_2+δ_ | 1.8–4.2 V | 550, 0.5 C | 125.5/ 71.0% | - | [S5] |
| 5 M NaFSI in DME | Na\|\|P2- Na_0.7_(Fe_0.5_Mn_0.5_)O_2_ | 1.5–4.3 V | 100, 0.5 C | About 110/ 82.1% | - | [S6] |
| **1.2 M NaTFSI in SUL:OTE (1:1 molar ratio) with 5 wt.% FEC** | **Na\|\|O3- NaNi_1/3_Mn_1/3_Fe_1/3_O_2_** | **2**–**4.2 V** | **300, 1 C** | **130.82/ 79.48%** | **No** | **This work** |
|  |  |  | **400, 2 C** | **116.39/ 81.15%** |  |  |

**Supplementary References**

1. J. Sung, N. Shaji, T. Kim, F. Jiang, M. Nanthagopal et al., The effect of nonflammable electrolyte on Cu-substituted P2-type layered cathode for high safety sodium-ion batteries. J. Power Sources **580**, 233266 (2023). https://doi.org/10.1016/j.jpowsour.2023.233266
2. Q. Liu, Y.-H. Feng, X. Zhu, M. Liu, L. Yu et al., Stabilizing cathode-electrolyte interphase by localized high-concentration electrolytes for high-voltage sodium-ion batteries. Nano Energy **123**, 109389 (2024). https://doi.org/10.1016/j.nanoen.2024.109389
3. P. Dai, C.-G. Shi, Z. Huang, X.-H. Wu, Y.-P. Deng et al., A new film-forming electrolyte additive in enhancing the interface of layered cathode and cycling life of sodium ion batteries. Energy Storage Mater. **56**, 551-561 (2023). https://doi.org/10.1016/j.ensm.2023.01.046
4. J. Fan, P. Dai, C. Shi, Y. Wen, C. Luo et al., Synergistic dual‐additive electrolyte for interphase modification to boost cyclability of layered cathode for sodium ion batteries. Adv. Funct. Mater. **31**(17), (2021). <https://doi.org/10.1002/adfm.202010500>
5. J. Lin, H. Peng, P. Huang, T. Naren, C. Liang et al., Electrically coupled electrolyte engineering enables high interfacial stability for high‐voltage sodium‐ion batteries. Adv. Funct. Mater. **33**(48), 2307061 (2023). <https://doi.org/10.1002/adfm.202307061>
6. J. Lee, Y. Lee, J. Lee, S. Lee, J. Choi et al., Ultraconcentrated sodium bis(fluorosulfonyl)imide-based electrolytes for high-performance sodium metal batteries. ACS Appl. Mater. Interfaces **9**(4), 3723-3732 (2017). <https://doi.org/10.1021/acsami.6b14878>
